# Multi-gene biomarkers reveal spatial organization and subpopulation-specific damage response in biliary epithelial cells

**DOI:** 10.1242/dev.205690

**Published:** 2026-10-12

**Authors:** Kendall G. Kanakanui, Fransky Hantelys, Hannah R. Hrncir, Sergei Bombin, Adam D. Gracz

**Affiliations:** ^1^ Graduate Program in Genetics and Molecular Biology, Emory University, Atlanta, GA 30322, USA; ^2^ Department of Cell Biology & Physiology, University of North Carolina at Chapel Hill, Chapel Hill, NC 27599, USA; ^3^ Department of Medicine, Division of Digestive Diseases, Emory University, Atlanta, GA 30322, USA; ^4^ Graduate Program in Biochemistry, Cell and Developmental Biology, Emory University, Atlanta, GA 30322, USA

**Keywords:** Biliary epithelial cell, Cholangiocyte, Liver, Cellular heterogeneity, scRNA-seq

## Abstract

Biliary epithelial cell (BEC) heterogeneity remains challenging to define. Here, we performed scRNA-seq on Sox9^EGFP+^ liver epithelium from mice subjected to bile duct ligation (BDL) and sham controls. We identified 4 BEC subpopulations in sham controls, each associated with specific enrichment of a minimal one or two gene biomarker (‘metagene’). Metagenes were validated by hybridization chain reaction fluorescence *in situ* hybridization, which also facilitated spatial analysis of intrahepatic bile ducts. BECs belonging to a given subpopulation were most likely to have neighbors of the same identity, forming homogenous cellular compartments within otherwise heterogeneous ducts. Subpopulation specific differential gene expression (DGE) analysis revealed that BDL decreases transcriptomic heterogeneity among BECs by suppressing subpopulation specific genes. BDL samples also included a proliferative subpopulation not found in controls, which contained populations enriched for three of the four metagenes. All BEC subpopulations were observed *in vitro* in monolayer cultures, where they clustered spatially with BECs of the same subtype. Together, our data demonstrate that metagene biomarkers facilitate spatial localization of BEC subpopulations *in situ*, identify subpopulation-specific injury responses and confirm that BEC heterogeneity is preserved *in vitro*.

## Teaser

**Summary:** Single-cell RNA-seq and multiplexed spatial RNA imaging define and validate a multi-gene biomarker panel for biliary epithelial cell subpopulations, describing cellular heterogeneity in homeostasis and following injury.

## INTRODUCTION

Biliary epithelial cells (BECs) line the intrahepatic and extrahepatic bile ducts (IHDBs and EHBDs, respectively), forming a continuous network that modifies bile and transports it from the liver to the digestive tract. Bile serves a crucial function in the break down dietary fats for nutrient absorption and the excretion of waste products, including excess cholesterol and xenobiotics. While BECs make up only 3-5% of the healthy liver by cell number, liver injury can induce a proliferative expansion of BECs, termed ductular reaction (DR). DR is thought to have both protective and maladaptive consequences, maintaining bile flow while also resulting in pro-fibrotic and pro-inflammatory signaling (Gupta et al., 2025; Hrncir et al., 2023). Overall, the vital role BECs play in regulating bile composition and bile flow is essential for proper liver function and to maintain organismal homeostasis (Alpini et al., 1988).

Despite evidence that subpopulations of BECs exhibit heterogeneous function in homeostasis and response to injury, BEC heterogeneity has remained challenging to define and assay. Classical models have defined BEC subpopulations relative to their localization in small ductules or large ducts (Alpini et al., 1997b; Glaser et al., 2009; Kanno et al., 2000). However, many of the biomarkers associated with small and large BECs are either not detectable or expressed broadly across BEC subpopulations in recent transcriptomic datasets (Andrews et al., 2022; Hrncir et al., 2025; MacParland et al., 2018; Tulasi et al., 2021). Single cell RNA-seq (scRNA-seq) has facilitated unbiased characterization of BEC heterogeneity but has yet to result in a definitive ‘ground truth’ understanding of BEC subpopulations consisting of broadly accepted cell types and their associated biomarkers. BECs are a relatively rare cell type in the liver and exhibit high cell-cell transcriptomic similarity to one another, resulting in under-representation in liver atlases and a lack of distinct ‘clusters’ in unbiased scRNA-seq analyses (MacParland et al., 2018; Pepe-Mooney et al., 2019; Planas-Paz et al., 2019; Pu et al., 2023). BEC subpopulations also lack well-defined, cell-type specific biomarkers, limiting *a priori* approaches to scRNA-seq analysis and high confidence identification of cell-cell heterogeneity. Previous studies have addressed this challenge by defining BEC subpopulations relative to proliferative status or in terms of gradients of gene expression (Andrews et al., 2022; Pepe-Mooney et al., 2019; Planas-Paz et al., 2019; Pu et al., 2023). For example, Yap signaling was observed to be active in all BECs but expressed at high and low levels in distinct BEC subpopulations (Pepe-Mooney et al., 2019). However, a reliance on transcriptome-level data limits consideration of BEC heterogeneity and subpopulation-specific responses to scRNA-seq assays.

Our lab has previously characterized BEC heterogeneity relative to expression of the transcription factor *Sox9*, using a Sox9^EGFP^ BAC transgenic reporter mouse (Tulasi et al., 2021). Sox9^EGFP^ is expressed in all BECs, but at distinct levels in BECs (termed GFP^low^ and GFP^high^), as well as a rare population of peribiliary hybrid hepatocytes (HybHeps, GFP^sublow^) (Font-Burgada et al., 2015; Tulasi et al., 2021). While the Sox9^EGFP^ allele is advantageous as a single biomarker approach for identifying transcriptomically distinct BEC subpopulations, whether it captures the full extent of BEC heterogeneity is unclear. In the present study, we sought to profile BEC heterogeneity and identify experimentally tractable biomarkers of BEC subpopulations by scRNA-seq.

## RESULTS

### Single cell transcriptomic profiling of BECs and hybrid hepatocytes

To produce a single cell atlas focused on BECs, we performed scRNA-seq on EGFP^+^ cells from both healthy and cholestatic Sox9^EGFP^ mouse livers. Mice were either subjected to bile duct ligation (BDL) or sham surgery and sacrificed after 7 days. We confirmed that EGFP marks BECs (EGFP+/EpCAM+) and HybHeps (EGFP^+^/EpCAM^−^) in both sham and BDL livers by immunofluorescence, as previously reported (Fig. 1A) (Tulasi et al., 2021). EGFP^+^ cells were isolated by fluorescence-activated cell sorting (FACS) and subjected to scRNA-seq using split-pool chemistry (Fig. S1A). Cells with fewer than 100 genes detected or with mitochondrial RNA content higher than 50% were excluded (Fig. S1B). Following QC filtering, our final dataset contained 6926 Sox9^EGFP+^ cells from two sham mice and 5282 Sox9^EGFP+^ cells from two BDL mice. Because no established data exist regarding the number of mouse BEC subpopulations, data were analyzed for optimal cluster number using clustree (Fig. S1C,D) (Zappia and Oshlack, 2018). To focus our analysis on epithelium, we excluded a cluster of contaminating endothelial cells, marked by the canonical endothelial marker *Pecam1*, as well as clusters containing fewer than 100 cells (Fig. S1E). We identified 13 high confidence clusters across sham and BDL samples (Fig. 1B). Interestingly, sham and BDL samples clustered separately, with individual biological replicates distributed uniformly, implying that cholestasis has a greater effect than subpopulation identity on BEC transcriptomic heterogeneity (Fig. S1F). To determine if differential clustering by injury status represents a batch effect, we performed Harmony integration using sample identity as the batch variable. Sham and BDL populations remained largely distinct following batch correction, even under a relatively stringent integration parameter (θ=3) (Fig. S1G). These results strengthen the conclusion that cholestatic injury induces greater transcriptomic changes in BECs than subpopulation identity.

**Fig. 1.**
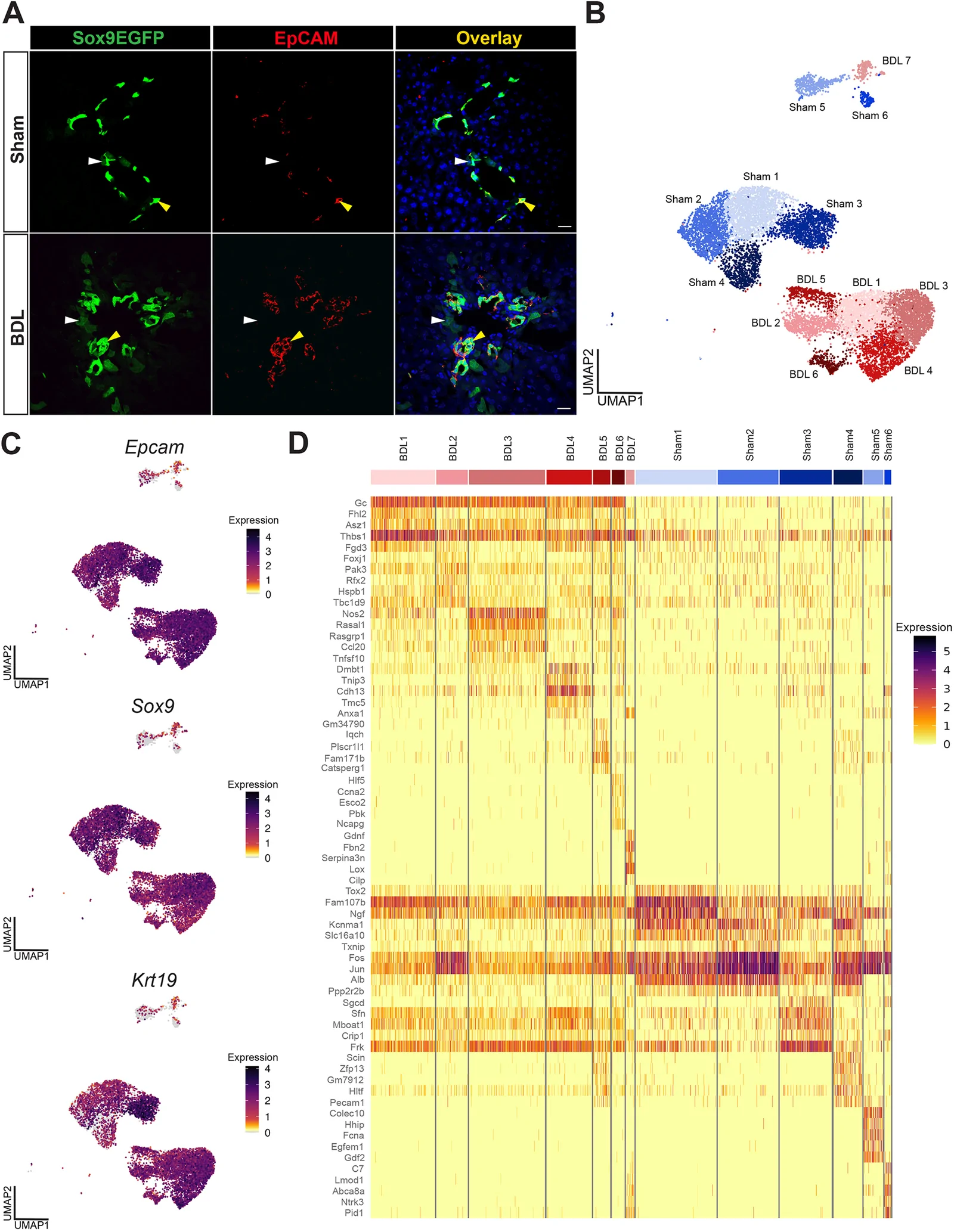
**scRNA-seq of Sox9^EGFP+^ cells captures biliary epithelial cells and hybrid hepatocytes in healthy and cholestatic livers.** (A) Immunofluorescence of sham-operated and 7 days post-bile duct ligation (BDL) Sox9^EGFP^ liver confirms that EGFP localizes to EpCAM^+^ biliary epithelial cells (BECs; yellow arrowheads) and EpCAM^−^ periportal HybHeps (white arrowheads). Scale bars: 10 μm. (B) EGFP^+^ cells can be classified into 13 transcriptomically distinct clusters, with differential clustering associated with sham versus BDL experimental groups. (C) Expression of canonical BEC markers *Epcam*, *Sox9* and *Krt19* confirms identity of BEC clusters (Sham 1-4, BDL 1-6). (D) The top 5 differentially expressed genes in BEC clusters (Sham 1-4, BDL 1-6) and HybHep clusters (Sham 5-6, BDL 7) exhibit varying levels of transcriptional heterogeneity across cell type and experimental group. Top genes in sham BEC clusters are broadly expressed across clusters, suggesting substantial transcriptomic similarity between individual BECs.

Since Sox9^EGFP^ is expressed in both BECs and HybHeps, we next sought to confirm the epithelial lineage identity of scRNA-seq clusters. First, we examined *Egfp*, which is known to be expressed highly in BECs and at low levels in HybHeps (Tulasi et al., 2021). *Egfp* was enriched in clusters Sham 1-4 and BDL 1-6, suggesting that these clusters represent BECs, while Sham 5, Sham 6 and BDL 7 represent HybHeps (Fig. S2A). Across all clusters, *Egfp* and endogenous *Sox9* exhibited a weak correlation that was characteristic of the expected scRNA-seq ‘drop out’ effect (Fig. S2B) (Kharchenko et al., 2014). To further examine BEC and HybHep cluster identities based on known Sox9^EGFP^ heterogeneity, we scored transcriptomic signatures of the three Sox9^EGFP^ populations (GFP^high^, GFP^low^ and GFP^sublow^) derived from published bulk RNA-seq (Tulasi et al., 2021). GFP^high^ and GFP^low^ signatures, which are associated with BEC populations, were enriched in Sham 1-4 and BDL 1-6, consistent with *Egfp* expression in these clusters (Fig. S2D,E). The HybHep GFP^sub^ gene signature was enriched in Sham 5, Sham 6 and BDL 7 (Fig. S2C). Together, these data identify BEC (Sham 1-4 and BDL 1-6) and HybHep (Sham 5, Sham 6 and BDL 7) clusters in our larger dataset.

Previous studies applying scRNA-seq to BECs note the following: (1) broad expression of universal BEC biomarkers across computationally identified clusters and (2) subpopulation-specific expression of genes associated with hepatocytes (Andrews et al., 2022; Hrncir et al., 2025; Planas-Paz et al., 2019). To examine this in our own dataset, we analyzed expression patterns of canonical BEC genes (*Epcam*, *Krt19* and *Sox9*) and canonical hepatocyte genes (*Hnf4a* and *Alb*). As expected, BEC genes were broadly expressed across BEC clusters and showed lower expression in HybHep clusters (Fig. 1C). *Krt19* was further enriched in Sham 3, consistent with earlier observations that the Sox9^EGFP-low^ subpopulation expresses elevated *Krt19* relative to other BECs (Fig. 1C and Fig. S2D) (Tulasi et al., 2021). Unexpectedly, *Hnf4a* and *Alb* exhibited higher expression in BEC clusters than in HybHep clusters (Fig. S3A). While it is possible that the presence of HybHeps in our preps resulted in the contamination of BECs with hepatocyte transcripts, this seems unlikely given: (1) the broad detection of *Hnf4a* and *Alb*, and (2) the presence of these and other hepatocyte-associated transcripts in other published scRNA-seq of mouse BECs (Planas-Paz et al., 2019; Pu et al., 2023). Hepatocyte biomarker expression was somewhat variable between BEC clusters, with decreased *Hnf4a* expression in Sham 2 and 4, and decreased *Alb* expression in BDL 3 relative to other BEC clusters (Fig. S3A). We also examined expression of reported small BEC and large BEC markers, which represent classical markers of BEC heterogeneity (Alpini et al., 2011, 1997b; Francis et al., 2026; Glaser et al., 2009; Luo et al., 2019). While the small BEC marker *Hrh1* was enriched in Sham 3 and BDL 3-4, other markers were either broadly expressed in BEC clusters or exhibited rare, scattered detection across clusters (Fig. S3B,C). Together, the expression of canonical BEC and hepatocyte genes reinforces the overall epithelial identity of clusters in our datasets and confirms previously noted transcriptomic similarities between BECs.

Previous studies have relied on enrichment of gene sets associated with proliferation, hepatocyte identity or Yap signaling to define differences between otherwise transcriptomically similar BEC subpopulations (Pepe-Mooney et al., 2019; Planas-Paz et al., 2019). To test if a similar approach could discriminate clusters in our dataset, we curated eight gene sets related to BEC specification and function from published data, including gene sets discriminating large ducts from small ductules (Table S1) (Coulouarn et al., 2008; Hrncir et al., 2025; Schaub et al., 2018; Villanueva et al., 2012). Gene sets for Yap, Notch and primary cilia were enriched in BEC clusters, whereas Hedgehog and ECM gene sets were enriched in HybHep clusters (Fig. S2F-J). Yap, Notch and TGF-β signaling were generally upregulated in BDL clusters relative to Sham clusters, supporting injury-induced activation of these pathways (Fig. S2F-G,K) (Planas-Paz et al., 2019; Seyhan et al., 2006; Zhang et al., 2016). Additionally, genes upregulated in large ducts versus small ductules were enriched in Sham 3 and BDL 3, suggesting that BEC identity associated with anatomical location in the IHBDs is maintained in both homeostasis and injury (Fig. S2L). While gene set enrichment analyses (GSEA) reveal subpopulation-specific differences that reinforce differences between BEC subpopulations, they mainly exhibit subtle relative enrichment or de-enrichment across BEC clusters. Further, because the gene sets examined here consist of 23-740 genes, they remain impractical as biomarkers of BEC subpopulations in assays other than scRNA-seq.

To pursue unbiased identification of individual genes that could serve as BEC subpopulation biomarkers, we performed differential gene expression between all clusters (Table S2). The top 5 differentially expressed genes (DEGs) from each cluster exhibited distinct expression between: (1) BEC and HybHep populations, and (2) sham and BDL populations (Fig. 1D). However, like previously examined gene sets associated with BECs, top DEGs for each BEC cluster exhibited either: (1) low coverage of individual cells within its cluster or (2) broad expression across other BEC clusters (Fig. 1D). Together, these findings demonstrate substantial transcriptomic differences between BECs and HybHeps, and BECs from healthy livers versus BECs from livers subjected to BDL. As with other published scRNA-seq datasets, our analyses also suggest that BEC subpopulations are highly similar at the transcriptomic level, complicating identification of subpopulation-specific biomarkers.

### Candidate metagenes define BEC subpopulations in homeostasis

To identify ‘ground truth’ biomarkers for BEC subpopulations, we reasoned that first defining BEC heterogeneity during homeostasis would facilitate identifying cognate subpopulations in BDL samples. Accordingly, we performed differential gene expression analysis on only sham controls (Table S3). We identified 1853 DEGs, with Sham 1- and Sham 2-associated DEGs exhibiting broad expression across all four clusters while Sham 3- and Sham 4-associated DEGs were more restricted to their respective clusters (Fig. 2A). GSEA revealed subpopulation-specific enrichment in gene sets consistent with BEC and/or epithelial function (Fig. 2B). Importantly, GSEA results for each cluster exhibited nonoverlapping significant hits, supporting the unique identity of the four putative BEC subpopulations in our dataset.

**Fig. 2.**
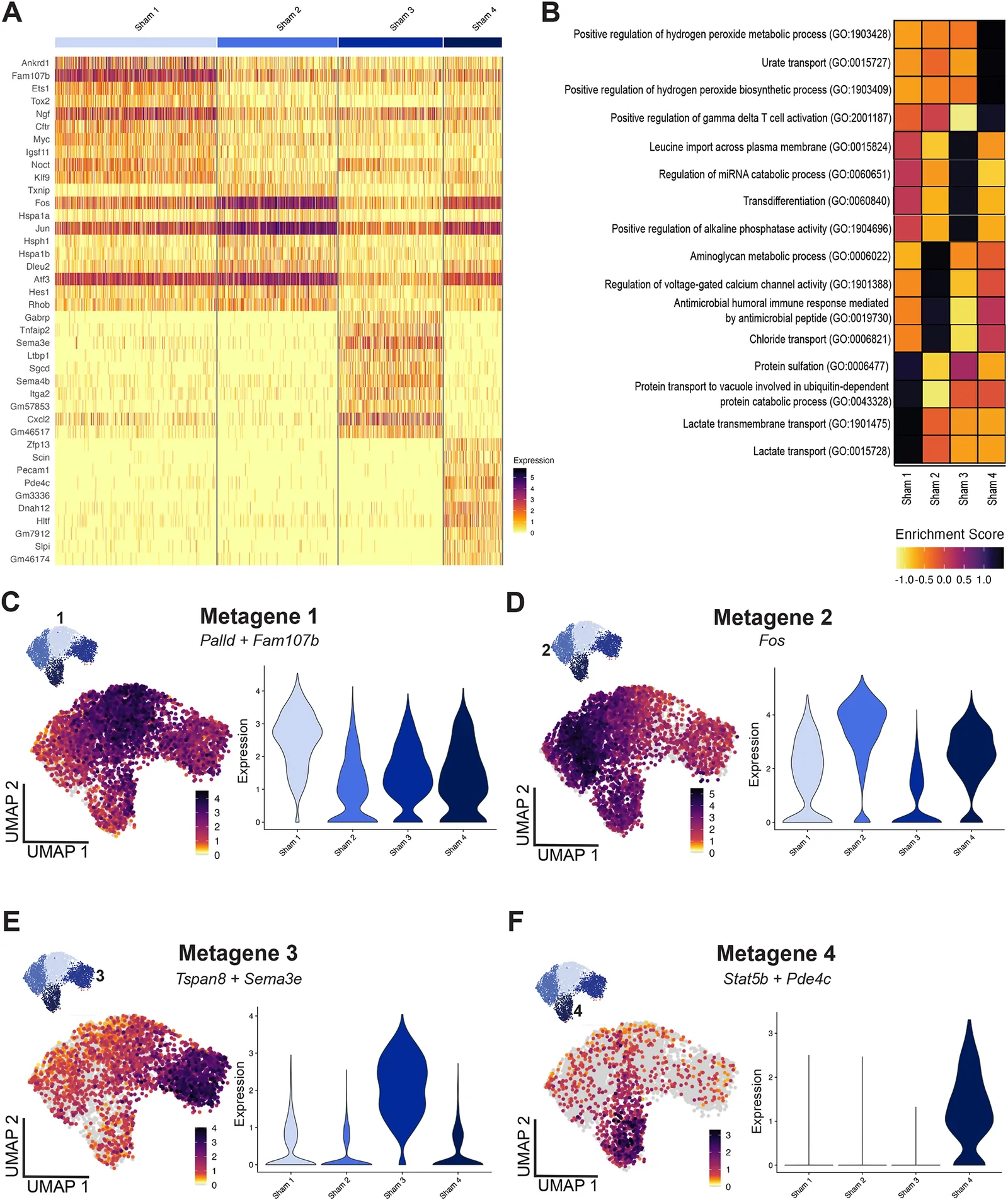
**Biliary epithelial cell subpopulations in sham-operated controls can be defined by the expression of ‘metagene’ biomarkers.** (A) The top 10 differentially expressed genes in Sham 1-4 support high transcriptomic similarities between biliary epithelial cells (BECs), based on broadly expressed genes. (B) GSEA suggests that sham BEC subpopulations exhibit different functional properties, supporting the unique identity of each cluster. (C-F) Differential expression of candidate metagenes across the four sham BEC subpopulations supports their potential as biomarkers of BEC heterogeneity. Each metagene demonstrates enrichment in its ‘on target’ cluster and de-enrichment in ‘off target’ clusters. (Scale represents normalized metagene expression.)

DEGs identified for Sham 3 and Sham 4 exhibited ‘binary’ expression, an important characteristic of potential biomarkers defined by enrichment in the ‘target’ cluster and simultaneous de-enrichment in all other clusters (Aevermann et al., 2021). However, DEGs identified for Sham 1 and Sham 2 did not exhibit binary expression, as many were also expressed at variable levels in Sham 3 and Sham 4 (Fig. 2A). To identify genes with binary expression that might not be represented in the top DEGs for each cluster, we applied NSForest, a previously published machine learning algorithm (Aevermann et al., 2021, 2018). NSForest identifies potential biomarkers in scRNA-seq data by scoring genes for both binary expression as well as expression in all cells within a defined cluster (Aevermann et al., 2021). We identified four minimal gene biomarkers composed of one or two genes associated with each subpopulation, which we termed ‘metagenes’. *Palld* and *Fam107b* (metagene 1) identify Sham 1, *Fos* (metagene 2) defines Sham 2, *Tspan8* and *Sema3e* (metagene 3) define Sham 3, and *Stat5b* and *Pde4c* (metagene 4) define Sham 4 (Fig. 2C-F). *Palld* encodes a cytoskeletal protein involved in actin organization and *Fam107b* encodes a nuclear protein likely involved in stress response (Boukhelifa et al., 2004; Nakajima et al., 2010). Metagene 2 consists only of *Fos*, which encodes a subunit of the transcription factor AP-1 and is associated with hepatocellular carcinoma (HCC) in humans (Hu et al., 2020). In metagene 3, *Tspan8* encodes a transmembrane protein important in lipid metabolism and *Sema3e* encodes a secreted class 3 semaphorin involved in several context-dependent processes, such as regulation of sinusoidal regeneration in liver fibrosis during liver regeneration (Alto and Terman, 2017; Hemler, 2005; Min et al., 2023; Yagai et al., 2014). Both *Tspan8* and *Sema3e* have been identified as markers for several cancers, and specifically *Tspan8* has shown to regulate the stemness of breast and pancreatic cancer cells (Fu et al., 2017; Planas-Paz et al., 2019; Wang et al., 2013; Yong et al., 2016). In metagene 4, *Stat5b* encodes a transcription factor with potential fibrotic and metabolic regulatory roles, and *Pde4c* encodes an enzyme that functions in cAMP signaling specifically associated with inflammation (Waddleton et al., 2008; Yu et al., 2010). *Tspan8* has been proposed to be a novel BEC marker, but to our knowledge, the genes comprising the metagenes have not been previously proposed as subpopulation-specific biomarkers for BECs (Planas-Paz et al., 2019).

We next compared the expression of all four metagenes across Sham BEC subpopulations. Only Metagene 4 exhibited striking binary expression (Fig. 2F). The other three metagenes had varying degrees of binary expression, with metagene 3 demonstrating the next most restrictive expression and metagene 1 demonstrating the broadest expression across BEC subpopulations (Fig. 2C-E). Interestingly, metagenes consisting of two genes (metagenes 1, 3 and 4) have a similar pattern of co-expression. One gene was highly expressed in its specific cluster (*Palld*, *Tspan8* and *Stat5b*), but also in other clusters (Fig. S4A,D,F). The other gene was more restricted to its specific cluster (*Fam107b*, *Sema3e* and *Pde4c*), but exhibited lower coverage of individual cells within its cluster (Fig. S4B,E,G). To further evaluate our marker selection strategy, we applied an alternative approach for detecting genes with binary expression. We computed the difference in the proportion of cells expressing a given gene in one sham BEC cluster compared to the other three sham BEC clusters (Δpct=pct1−pct2). Using this approach, we identified the top 10 genes for each sham BEC cluster based on ranked Δpct (Table S4). As metagenes 1 and 2 exhibited the least binary expression patterns, we specifically examined alternatives for the broadly expressed gene in each (*Palld* and *Fos*, respectively) and generated new composite plots based on the highest Δpct value (Fig. S4H-K). Relative to NSForest-selected candidates, metagenes containing alternative genes (*Ets1* and *Myc* for metagene 1 and *Txnip* for Metagene 2) demonstrated greater enrichment within the ‘target’ cluster, but reduced specificity (Fig. S4H,J,K). *Ngf*+*Fam107b* showed comparable enrichment to *Palld*+*Fam107b* for metagene 1 (Fig. S4I). For this reason, we proceeded to apply NSForest-selected metagenes throughout our study. As a panel, these metagenes represent potential biomarkers for identifying BEC subpopulations, which can be identified by: (1) enrichment of the cluster specific metagene and (2) the de-enrichment of metagenes associated with other BEC subpopulations.

### Spatial validation of BEC metagenes identifies homogenous cellular neighborhoods within heterogeneous ducts

To test whether candidate metagenes could function as BEC subpopulation biomarkers *in situ*, we designed a 7 gene panel for hybridization chain reaction (HCR) fluorescent *in situ* hybridization (FISH) and applied this to liver tissue sections from wild-type C57Bl/6 mice (Fig. 3A) (Schulte et al., 2024). Because HCR FISH allows the relative quantification of gene expression, BECs were segmented based on EpCAM signal and scored based on relative metagene expression to derive a subpopulation assignment (Fig. 3B and Table S5). Each BEC demonstrated clear enrichment of a single metagene and de-enrichment of the other three metagenes, as observed in scRNA-seq analysis. We quantified 243 BECs across 23 fields from three biological replicates, and identified 24.69% of BECs enriched for metagene 1, 23.87% of BECs enriched for metagene 2, 28.81% of BECs enriched for metagene 3 and 22.63% of BECs enriched for metagene 4. We compared the proportion of each BEC subpopulation identified in HCR FISH data to proportions from scRNA-seq and observed high concordance between the two detection methods (Fig. 3C). Notably, differences in proportions of each BEC subpopulation in HCR FISH and scRNA-seq amounted to less than 12% (Fig. 3C). These results suggest that *in situ* quantification of candidate metagenes is sufficient to discern BEC subpopulations identified by scRNA-seq. To simplify discussion of BEC subpopulations throughout the rest of this manuscript, each scRNA-seq identified cluster will be referred to by its corresponding Metagene number (for example, BECs enriched for metagene 1 are referred to as ‘BEC-I’).

**Fig. 3.**
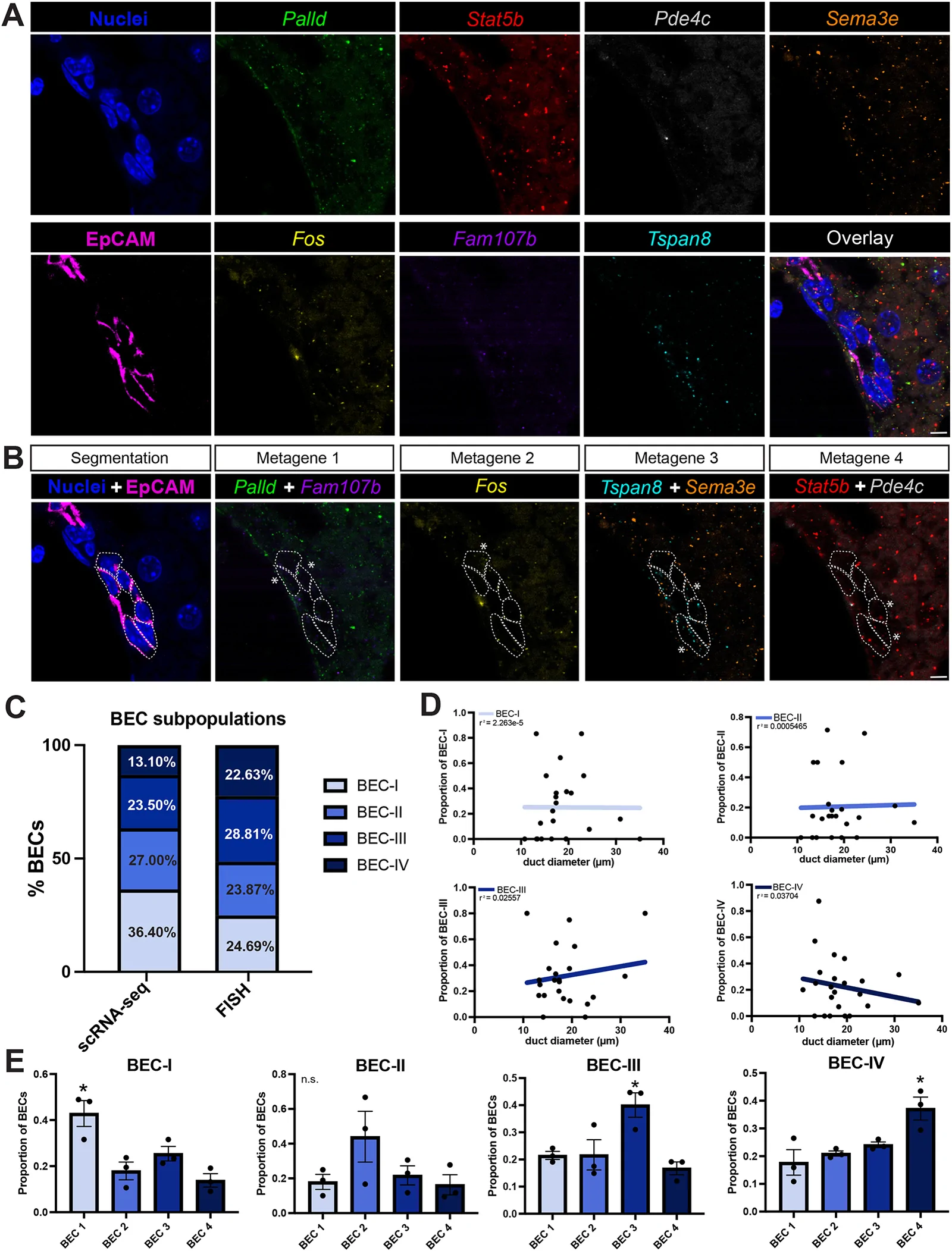
**Hybridization chain reaction fluorescent *in situ* hybridization for candidate metagenes validates biliary epithelial cell subpopulations *in vivo* and provides spatial context for biliary epithelial cell heterogeneity.** (A) Validation of a 7-plex hybridization chain reaction fluorescent *in situ* hybridization (HCR FISH) panel (*Palld*, *Stat5b*, *Pde4c*, *Sema3e*, *Fos*, *Fam107b* and *Tspan8*) for metagene detection in liver tissue from wild-type C57Bl/6 mice. (B) Biliary epithelial cell (BEC) identification and segmentation by EpCAM facilitates metagene quantification and classification of individual BECs by subpopulation identity (dashed lines represent BECs quantified; asterisks mark the BEC identity within each metagene-specific image field). (C) The proportions of each BEC subpopulation identified by HCR FISH are similar to proportions in scRNA-seq data. (D) Quantification of BEC identity relative to IHBD diameter reveals a trend for BEC-III localization to larger ducts and BEC-IV localization to smaller ducts (*n*=3). (E) Quantification of neighboring cell identities reveals BECs are likely to have neighboring BECs of the same subtype (*n*=3). n.s. not significant; *P<0.05 [simple linear regression (D); one-way ANOVA (E)]. Data are mean±s.e.m. Scale bars: 5 μm.

Next, we sought to leverage *in situ* application of the metagene panel to gain insight into the spatial organization of BEC subpopulations within IHBDs. Previous studies have suggested that BEC size correlates with IHBD size, as defined by duct diameter, and that both are linked to BEC identity (Alpini et al., 1997a, 1996; Ueno et al., 2003). Comparison of BEC area with both its corresponding duct diameter and subpopulation identity revealed no correlation, with small and large BECs present across ducts of varying sizes and among all subpopulations (Fig. S5C,D). Consistent with these findings, every duct analyzed contained at least two BEC subpopulations, suggesting that no single BEC identity corresponds to a specific IHBD size (Fig. S5E). While correlation between BEC subpopulation and IHBD size did not reach significance, BEC-III exhibited a weak correlation with larger ducts and BEC-IV with smaller ductules (Fig. 3D). This result is consistent with our previous studies demonstrating an increased proportion of Sox9^EGFP-low^ BECs in large IHBDs, as both GFP^low^ and BEC-III are enriched for *Krt19* (Fig. 1C and Fig. S2D) (Tulasi et al., 2021). The enrichment of GFP^low^ and large duct gene signatures in BEC-III versus BEC-I, BEC-II and BEC-IV are also supportive of BEC-III being more represented in larger IHBDs (Fig. 3D and Fig. S2D). To further examine the spatial organization of BECs, we quantified the subpopulation identity of neighboring cells for each individual BEC. BECs of each subpopulation were most likely to have a neighboring BEC of the same subpopulation, with the exception of BEC-II, which exhibited a similar trend that did not reach significance (Fig. 3E). Spatial data derived from HCR FISH collectively demonstrate that BEC subpopulations are distributed throughout IHBDs, with BEC-III enriched in larger ducts. Additionally, these data reveal that BECs are localized to relatively homogenous epithelial ‘neighborhoods’ within heterogeneous IHBDs.

### Metagenes facilitate identification of subpopulation-specific responses to cholestatic injury

A challenge presented by our initial scRNA-seq analysis was the inability to confidently match Sham clusters with their corresponding BDL cluster due to the substantial transcriptomic differences between each experimental group (Fig. 1B and Fig. S1G). Additionally, BDL BECs exhibited two additional clusters, further complicating the subpopulation identification across experimental conditions. Together, these technical challenges hindered our ability to examine BEC subpopulation-specific gene expression changes. We reasoned that we could use metagene enrichment to identify Sham and BDL clusters of the same BEC subpopulation identity. We first examined the enrichment of metagenes 1-4 in BDL samples and found that metagenes 1, 2 and 4 were enriched in a single BDL cluster (Fig. 4A). Interestingly, metagene 3 was enriched in both BDL 3 and BDL 4 (Fig. 4A and Fig. S6A). To characterize the transcriptomic differences between these subpopulations, we performed differential gene expression analysis and identified 447 DEGs (Table S6). GSEA revealed that BDL 3 is enriched for inflammation-associated gene sets, while BDL 4 is enriched for terms related to translation and ribosome assembly (Fig. S6C). Despite significant gene expression differences, the shared enrichment of metagene 3 in both clusters implies that they may have derived from BECs represented by Sham 3 (Fig. S6A-C). Therefore, in line with our goal to compare uninjured control BEC subpopulations with their cognate injured populations, we merged BDL 3 and BDL 4 into a single cluster for downstream analyses. Therefore, in line with our goal to compare uninjured control BEC subpopulations with their cognate injured populations, we merged BDL 3 and BDL 4 into a single cluster for downstream analyses. To keep subpopulation nomenclature consistent, we relabeled BDL clusters to match their metagene number (Fig. 4B). The percentage of cells in each subpopulation following BDL shifted in comparison to sham, with an increase in BEC-III (+28.37%) and concomitant decreases in BEC-I (−9.04%), BEC-II (−13.61%) and BEC-IV (−5.72%) (Fig. 4C).

**Fig. 4.**
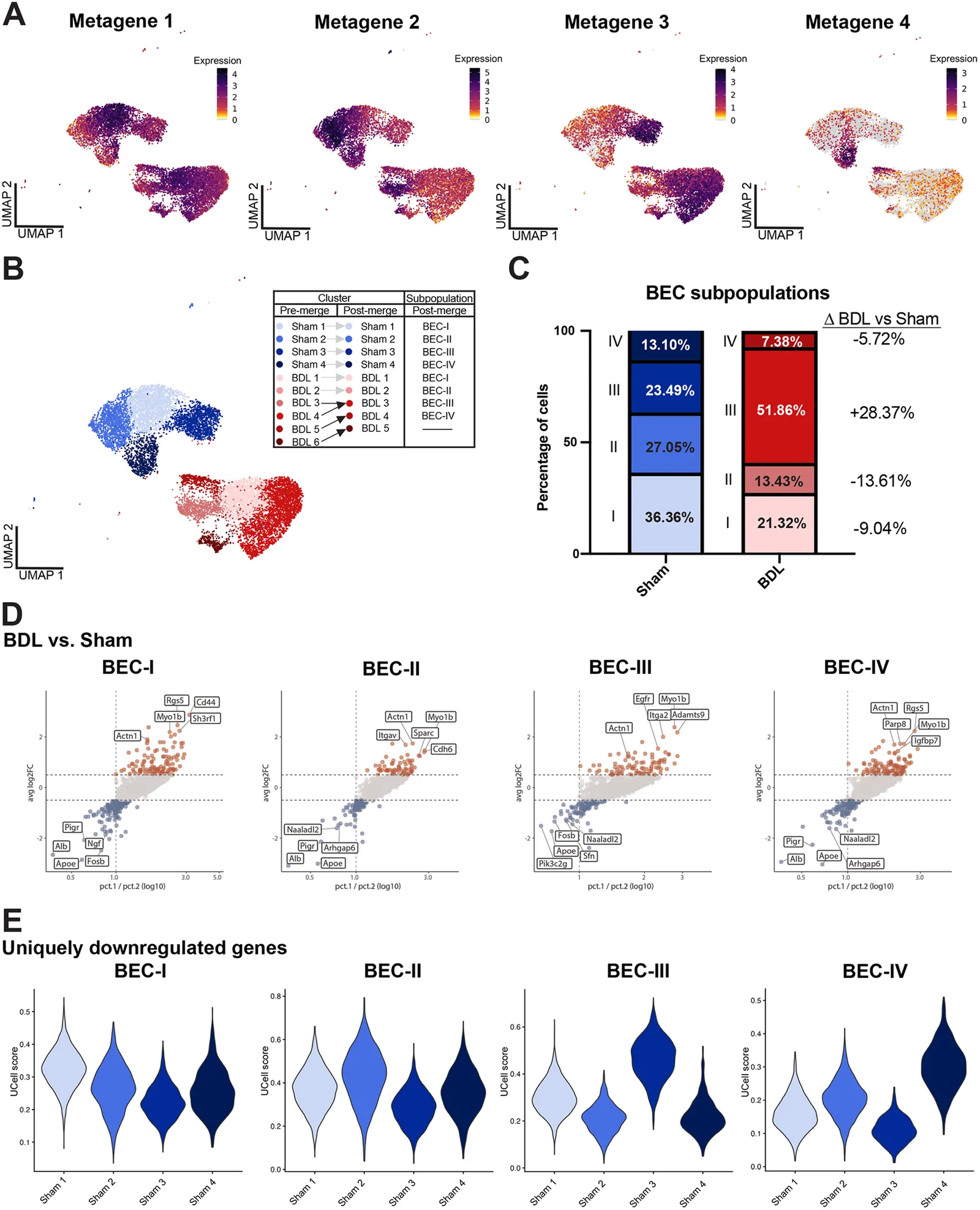
**Analysis of metagene-matched sham and bile duct ligation clusters reveals shared and subpopulation-specific responses to cholestasis in biliary epithelial cells.** (A) Metagene expression facilitates identification of biliary epithelial cell (BEC) subpopulations in bile duct ligation (BDL) samples that correspond to subpopulations identified in sham controls. Metagene 1 matches BEC-I to BDL 1, metagene 2 matches BEC-II to BDL 2, metagene 3 matches BEC-III to both BDL 3 and BDL 4, and metagene 4 matches BEC-IV to BDL 5. (B) BEC clusters were reassigned based on metagene enrichment (Sham 1-4 and BDL 1-5), where BDL 3 and BDL 4 merge into one cluster representing BEC-III, and BDL 5 is relabeled BDL 4 to represent BEC-IV. (C) BDL is associated with a change in BEC proportions by subpopulation, with an increase in BEC-III and corresponding decrease in all other subpopulations. (D) Differential gene expression analysis comparing each BEC subpopulation identifies significant upregulated and downregulated genes in BDL. Notably, genes associated with cell adhesion and cell locomotion are upregulated across all BEC subpopulations (*Actn1* and *Myo1b*), revealing a shared response to cholestasis. (E) Genes downregulated by BDL in a single BEC subpopulation are specifically enriched in that subpopulation in sham controls, suggesting that BDL suppresses subpopulation-specific transcriptomic identity in BECs.

We performed differential gene expression analysis comparing each BDL subpopulation to its respective sham subpopulation, using metagene enrichment as a determinant of BEC identity (Tables S7-S10). We identified 539 DEGs across all four subpopulation comparisons, of which 293 genes were shared between two or more comparisons (Fig. S7A). Based on the top 10 upregulated pathways by GSEA, terms related to cell adhesion, locomotion and motility, and cell organization were shared across all pairwise subpopulation comparisons (Fig. S7B). Examining the top 15 significantly upregulated genes for each subpopulation comparison, two genes were shared across all four comparisons (*Myo1b* and *Actn1*) (Fig. 4D). Both *Myo1b* and *Actn1* have been shown to be major regulators of adhesion and migration (Diaz-Valencia et al., 2022; Wang et al., 2023). Together, these data reveal BECs respond globally to cholestasis by upregulating gene expression associated with cellular adhesion and migration. Interestingly, the top 10 downregulated pathways in each of the pairwise comparisons had no terms shared across all four subpopulations (Fig. S7B). We hypothesized that BDL might be inducing injury-responsive genes broadly across BEC subpopulations while simultaneously repressing subpopulation-specific genes. To test the latter hypothesis, we examined expression of the four sets of uniquely downregulated DEGs in pairwise ‘Sham versus BDL’ comparisons across all BEC clusters. Because DE analyses of scRNA-seq can be underpowered, we first confirmed that each gene set was uniquely downregulated in its respective BDL cluster, validating differential gene expression analysis (Fig. S7C). Strikingly, each gene set was also enriched in its respective Sham subpopulation relative to all other Sham subpopulation (Fig. 4E). Taken together, these data support a model whereby BDL induces gene expression programs consistent with cellular adhesion and migration across all BEC subpopulations, while simultaneously downregulating subpopulation-specific genes.

In addition to the four metagene-defined BEC subpopulations in BDL, we observed a fifth cluster (BDL 5) marked by robust expression of several cell cycle genes (*Mki67*, *Cdc20* and *Cdk1*) (Fig. 5A). During homeostasis, ∼1-5% of BECs are proliferative by KI-67 staining (Masyuk et al., 2002). Following injury, IHBDs undergo ductular reaction marked by significantly increased BEC proliferation, consistent with the proportions of *Mki67^+^* cells in our dataset (Fig. 5B). Studies focused on BEC proliferation in response to ductular reaction found that BECs distributed throughout the IHBDs are capable of proliferating, but did not determine whether this response is restricted to a single BEC subpopulation (Kamimoto et al., 2016). Therefore, we sought to test which BEC subpopulations contribute to proliferation by analyzing the expression pattern of the four metagenes in BDL-5. We first subclustered BDL-5 into three clusters (C1-3) based on clustree analysis (Fig. 5C and Fig. S8). Metagenes 1-3 exhibited enrichment in a single subcluster, while metagene 4 was not reliably expressed in BDL-5 (Fig. 5D). These data suggest that BEC I-III are capable of entering the cell cycle following injury and that proliferative responses are not restricted to a single BEC subpopulation.

**Fig. 5.**
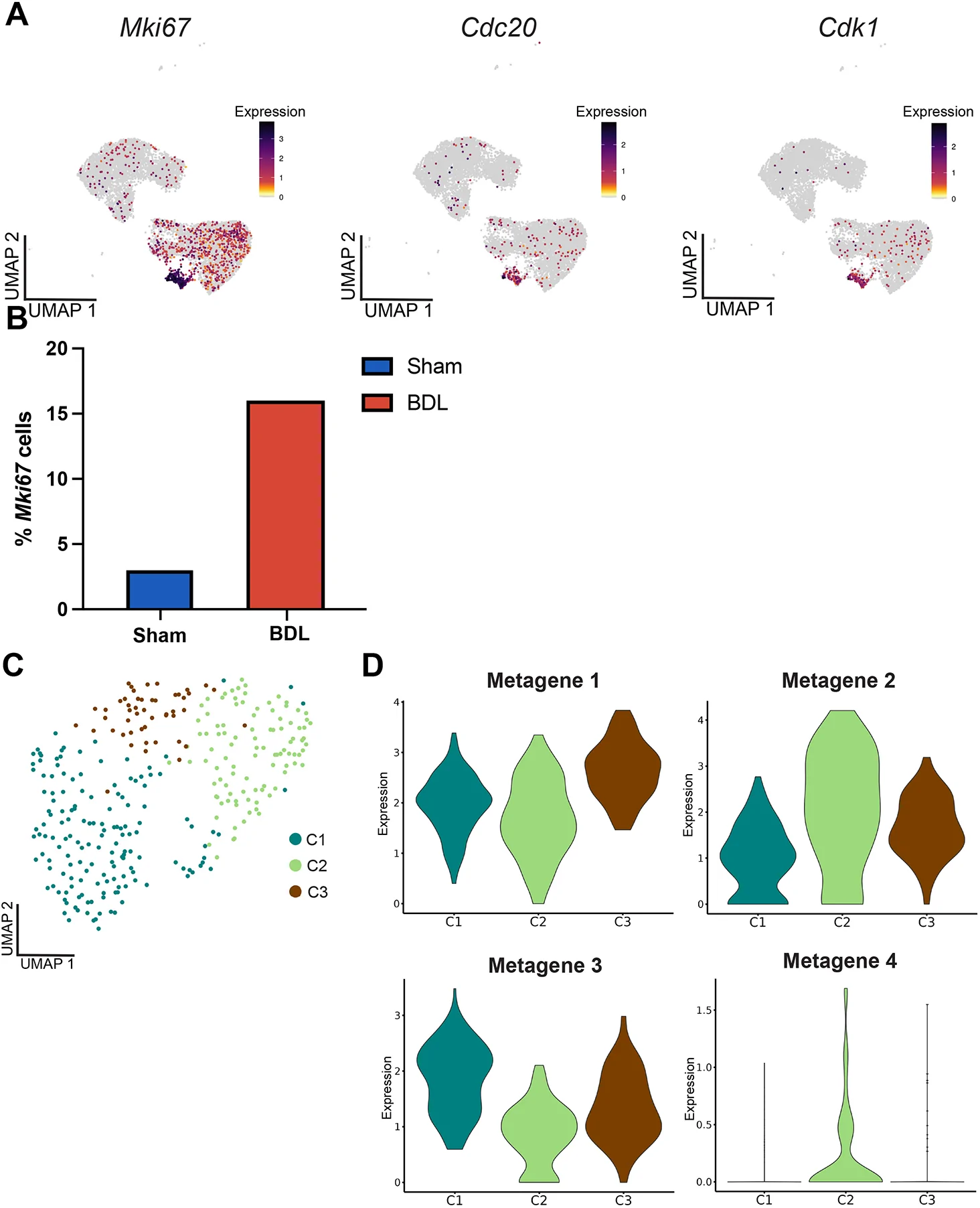
**Metagene expression defines biliary epithelial cell heterogeneity in bile duct ligation-specific, highly proliferative biliary epithelial cells*.*** (A) Cell cycle-associated genes *Mki67*, *Cdc20* and *Cdk1* are expressed throughout the Sham and bile duct ligation (BDL) subpopulations, but are strongly upregulated in a fifth subpopulation (BDL 5) found only following BDL. (B) The proportion of biliary epithelial cells (BECs) expressing *Mki67* in Sham versus BDL increases over threefold following injury, supporting significant proliferation induced by BDL. (C) Subclustering of BDL 5 reveals three distinct subclusters (C1-C3). (D) Differential enrichment of metagene 1 in C3, metagene 2 in C2 and metagene 3 in C1 suggest that BEC-I, BEC-II and BEC-III upregulate cell cycle genes in response to BDL. Low expression of metagene 4 across the same clusters suggests that BEC-IV are incapable of proliferating following injury or represent a transcriptomic program that is repressed following BDL.

### BEC metagene biomarkers and spatial neighborhoods are preserved *in vitro*

To explore *in vitro* BEC heterogeneity relative to metagenes, we isolated IHBD fragments from wild-type mice and cultured the cells to confluency. We then applied our 7-probe HCR FISH panel and quantified 1578 cells in total (Fig. 6A,B). BEC subpopulation proportions *in vitro* resembled those observed *in vivo*, with nominal differences (1% decrease in BEC-I; 3% decrease in BEC-II; 0.4% decrease in BEC-III; 4% increase in BEC-IV) (Fig. 6C). As determined in our *in vivo* analyses, we also found no significant correlation between cell size and BEC subpopulation identity *in vitro* (Fig. S9). Next, we asked if the spatial organization of BEC subpopulations observed *in vivo* is preserved in monolayer cultures *in vitro*. Qualitative analysis showed that cells of each BEC subpopulation localized near cells of the same subpopulation, forming homogeneous neighborhoods similar to those observed *in vivo* (Fig. 6D). Together, these results demonstrate that metagene expression is preserved *in vitro* and continues to identify distinct subpopulations of BECs. Further, the spatial organization of BECs of the same subpopulation in discrete clusters in the absence of a mesenchymal niche may suggest that BEC heterogeneity is regulated in an epithelial-autonomous manner.

**Fig. 6.**
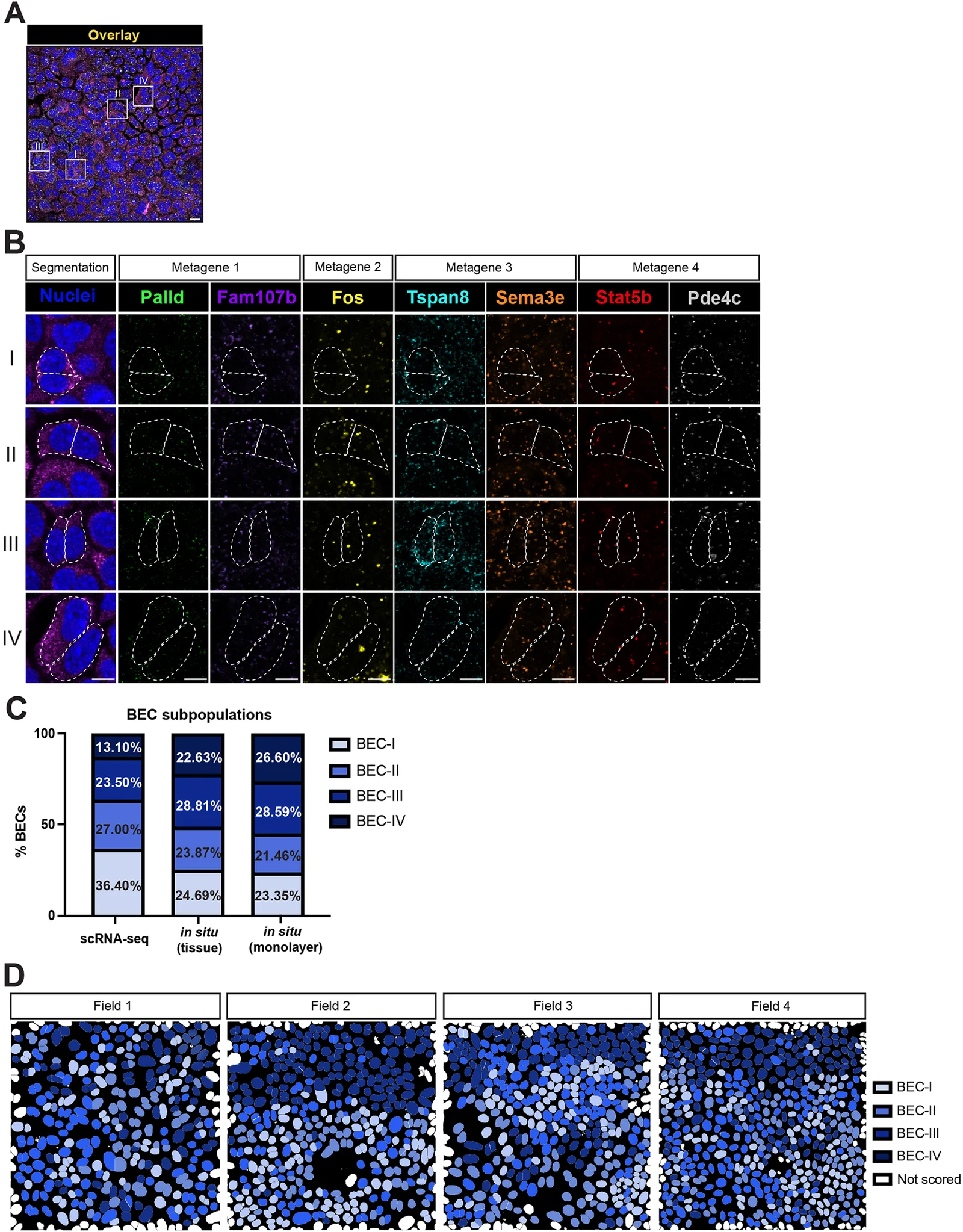
**Metagene-defined biliary epithelial cell heterogeneity is maintained *in vitro*.** (A) Representative composite overlay of a 7-plex hybridization chain reaction fluorescent *in situ* hybridization (HCR FISH) imaging panel in biliary epithelial cell (BEC) monolayers expanded for 7 days *in vitro* (white squares indicate an area of metagene enrichment, shown at higher magnification in B. Scale bar: 20 μm. (B) High-magnification single-channel images reveal metagene enrichment patterns that define BEC subpopulations (each row corresponds to a BEC subpopulation; dashed lines outline cells with the same subpopulation identity). Scale bars: 10 μm. (C) The proportion of BEC subpopulations in BEC monolayers are similar to observed proportions in tissue and quantified from scRNA-seq. (D) Spatial maps of the four imaging fields representing the 1578 cells analyzed illustrate the organization of BEC subpopulations within monolayer cultures (*n*=2). Cells belonging to the same BEC subpopulation preferentially localize near one another, forming homogeneous spatial neighborhoods that recapitulate the spatial patterning observed *in vivo*.

## DISCUSSION

Despite the power of scRNA-seq to define cellular heterogeneity across many tissue types and pathophysiological contexts, a clear consensus on intrahepatic BEC heterogeneity and subpopulation-specific biomarkers has remained elusive. Inherent technical challenges, including the relative scarcity of BECs versus other major hepatocellular lineages and the high transcriptomic similarities between BECs, have stymied these efforts (MacParland et al., 2018). In this study, we first sought to identify BEC subpopulations and their associated biomarkers by applying a previously characterized Sox9EGFP allele. By FACS-purifying *Sox9*^+^ cells, we generated a scRNA-seq dataset focused specifically on BECs and HybHeps (Tulasi et al., 2021). Notably, we identified two subpopulations of HybHeps at homeostasis. These appear to adopt a more homogenous transcriptome – represented by a single population – following BDL. Though outside of the scope of the current manuscript, our results suggest a previously unappreciated degree of heterogeneity in *Sox9*^+^ peribiliary hepatocytes.

Because standard analysis of DEGs failed to identify cluster-specific BEC biomarkers, we pursued a metagene biomarker approach based on: (1) the co-expression of multiple genes in ‘on-target’ clusters and (2) the de-enrichment of ‘off-target’ metagenes in ‘on-target’ clusters. Metagenes have been applied to represent transcriptome level patterns of gene expression via a limited number of gene biomarkers in microarray analysis and are seeing continued application to advanced multi-omics and spatial transcriptomics assays (Zhang et al., 2025). We propose that metagenes can be used to identify four distinct subpopulations of BECs in mouse IHBDs. Applications of these metagenes could range from spatial localization of BECs, as in our HCR FISH assays, to identification of BEC subpopulations across experimental groups, as demonstrated in our Sham versus BDL analysis.

The most comprehensive hepatobiliary atlases to date have been generated from human liver tissue. These efforts have resulted in the identification of 6 BEC subpopulations [named cholangiocyte (Chol)-1 to Chol-6], first in primary tissue by single nucleus RNA-seq (snRNA-seq) and more recently by scRNA-seq in organoids grown in refined media conditions that increase BEC heterogeneity *in vitro* (Andrews et al., 2022; Bregante et al., 2026). Our results differ from human data in that we were unable to identify the previously described proliferative/progenitor-like and hepatocyte-like populations in homeostatic control mouse liver (Andrews et al., 2022). Rather, we observe broad expression of hepatocyte genes *Hnf4a* and *Alb* across all BECs and a distinct proliferative cluster that emerges only after BDL. These results are unlikely to be due to under clustering, as our clustree analyses suggest that increasing clustering resolution to generate 6 Sham BEC subpopulations would be consistent with over clustering (Zappia and Oshlack, 2018). Our results resemble other scRNA-seq data of mouse BECs, where proliferative and hepatocyte-like clusters are reported in context of injury (Planas-Paz et al., 2019; Pu et al., 2023). These data may suggest that additional subpopulations in human atlases reflect fundamental differences between mouse and human biology. Alternatively, samples collected for human transcriptomic atlases may encompass a broader range of pathophysiological states, as human tissue acquisition for research is typically associated with underlying liver disease. It is notable that we initially identified 6 BEC clusters in our BDL samples, which we reduced to 5 by merging two clusters exhibiting similar enrichment of metagene 3. Because computational identification of cellular subpopulations in scRNA-seq data analysis is susceptible to over-clustering, we prioritized a conservative approach to identify BEC subpopulations that would allow us to compare Sham and BDL conditions. We also reasoned that, given the lack of substantial hepatocyte-to-BEC transdifferentiation following BDL, all BDL BEC subpopulations were most likely to be derived from BEC subpopulations present in sham controls (Yanger et al., 2013). Therefore, shared enrichment of metagene 3 could suggest that the BDL clusters we chose to merge were both derived from BEC-III. However, definitively proving precise relationships between BEC subpopulations in homeostasis and following injury will require advanced lineage tracing techniques and is beyond the scope of the current study. Another important consideration is whether other models of cholestatic or hepatocellular liver injury will induce biological responses conducive to application of the metagenes described here, or if biliary heterogeneity varies substantially in different contexts. Future studies may be able to further resolve BEC transcriptomic heterogeneity in different injury and disease models, using the metagene-defined subpopulations identified here as a foundation.

Another technical consideration is that our dataset was generated using split-pool based scRNA-seq technology, enabling whole-transcript coverage (Rosenberg et al., 2018). While *in situ* validation of candidate metagene biomarkers strongly supports the BEC subpopulations proposed here, whole transcript and 3′ end-counting scRNA-seq methods are known to yield subtle differences in transcript representation (Ma et al., 2019). Gene expression may also be an inherently poor readout of cell-cell heterogeneity in BECs. In this study and earlier work, we observe *Hnf4a* expression throughout BECs, but with little-to-no associated expression of HNF4A protein (Tulasi et al., 2021). Similarly, *Krt19* is enriched in a subpopulation of BECs while its protein product is ubiquitously expressed across all intrahepatic bile ducts with no apparent cell-cell difference in level (Tulasi et al., 2021). On one hand, potential discrepancies between BEC gene and protein expression could complicate efforts to easily identify subpopulation-specific protein biomarkers using transcriptomic data. However, it remains possible that protein expression patterns are more subpopulation specific than gene expression patterns in BECs. While single cell proteomics remains an emerging field, substantial recent advances may soon facilitate its broader application (Bubis et al., 2025; Ye et al., 2025). Future efforts integrating multiple datasets and methodologies may hold potential to yield improved BEC subpopulation biomarkers.

An unresolved issue in the field is whether BECs even exist as well-defined and ‘fixed’ subpopulations. Evidence from prior scRNA-seq studies comparing regional heterogeneity across the biliary tract supports a model where BECs are instead defined by highly plastic transcriptomic ‘states’. While regional differences between primary gallbladder, EHBD and IHBD epithelium were evident by scRNA-seq, BECs isolated from three-dimensional organoids of the same regions demonstrated more homogenous transcriptomes (Rimland et al., 2021; Sampaziotis et al., 2021). More recent studies have shown that conventional human BEC organoid cultures are similarly homogenous at the single cell level, but that refined culture conditions can restore transcriptomic heterogeneity observed *in vivo* (Bregante et al., 2026). This suggests that BEC heterogeneity *in vitro* is dependent on the proper milieu of extrinsic signaling present in culture conditions and is consistent with the known impact of culture media composition on cell fate in mouse and human intestinal organoids (Fujii et al., 2018; Yin et al., 2014). Our BDL results are also supportive of transcriptomic plasticity in BEC subpopulations, in that subpopulation-specific genes are downregulated while upregulated genes are most likely to be shared across multiple BEC subpopulations. However, despite larger transcriptomic changes, metagene expression remains useful in discriminating BEC heterogeneity in BDL. Similarly, our *in vitro* data demonstrate that BEC heterogeneity relative to metagene enrichment is preserved. While a limitation of the present study is that the full extent of transcriptomic heterogeneity in these conditions remains unknown, our results suggest that BEC metagenes may prove useful for assessing cellular heterogeneity in different contexts where pursuing scRNA-seq is either impossible or undesirable.

Inferring the functional and mechanistic impact of BEC heterogeneity remains a significant challenge. With the lack of robust biomarkers for BEC subpopulations coupled with the notable transcriptomic plasticity of BECs across physiological contexts, efforts are complicated when defining discrete BEC identities. Rather than representing fixed cell types, BEC subpopulations may instead reflect dynamic transcriptional states that are shaped by local signaling environments. With our approach, metagenes offer a framework to define core BEC subpopulation identities despite ongoing transcriptional remodeling. By enabling consistent identification of BEC subpopulations across conditions, metagenes may facilitate optimization of culture conditions to enrich for specific BEC states, providing a tractable platform for future work focused towards subpopulation-specific functional assays.

## MATERIALS AND METHODS

### Mice

Development of Sox9^EGFP^ mice is described by Gong et al. (2003). The *Egfp* transgene was maintained at heterozygosity on the C57Bl/6 background. Sox9^EGFP^ mice were phenotyped visually by EGFP signal in the base of the hair follicle in the tail clipping using an epifluorescent microscope. All experiments were performed on adult mice of both sexes between 6 and 14 weeks of age.

For surgical procedures, Emory veterinarian staff performed both bile duct ligation and sham surgery as previously reported (Tag et al., 2015). Briefly, Sox9^EGFP+^ mice were anesthetized with 1-3% isoflurane in 100% oxygen and underwent a ∼2 cm sterile midline laparotomy. For BDL, the liver was gently elevated, and the gut retracted caudally using a sterile swab with sterile 0.9% saline, exposing the common bile duct. Dissection of the common bile duct was performed from the portal vein with microserrated forceps and ligated using nonabsorbable 5-0 polyester suture, followed by a second cranial ligation to ensure complete occlusion. The peritoneal cavity was rinsed with sterile 0.9% saline, and the peritoneum and skin were closed separately with running 6-0 nonabsorbable monofilament sutures. Mice recovered on heating pads and were monitored twice daily for 2 days and then daily thereafter. No adverse effects or unexpected mortality occurred. Animals were euthanized 7 days post-surgery and processed for either histology (*n*=3 per condition) or FACS (*n*=2 per condition) as described below. All animal procedures were reviewed and approved by the Institutional Animal Care and Use Committee (IACUC) as Emory University and UNC.

### Perfusion and tissue processing for histology

Tissues were perfused and fixed by intracardiac perfusion with ice-cold PBS followed immediately by freshly-made, ice-cold 4% paraformaldehyde (PFA; Thermo Fisher, 41678-5000) using a peristaltic pump (Kamoer DIpump550). Whole-liver lobes were dissected out and dropped into ice-cold 4% PFA and stored overnight at 4°C. Tissues were transferred into 30% sucrose and stored overnight at 4°C. Individual lobes were embedded into Tissue-Tek Optimal Cutting Temperature media (Sakura Finetek), frozen down on dry ice and stored at −80°C.

### Immunofluorescence on tissue sections

For conventional histology, frozen tissue samples were sectioned at 10 μm thickness (Leica, CM1860) and air-dried at room temperature for 3 h. Sections were rehydrated in PBS for 3 min and permeabilized using 0.3% Triton X-100 (Sigma, T8787-250ML) in PBS for 20 min, followed by an incubation in 5% normal donkey serum (NDS; Jackson Immuno, 017-000-121) for 45 min. Primary antibody diluted in PBS was applied and incubated overnight at 4°C. The following day, sections were rinsed three times in PBS (5 min each) and incubated with fluorophore-conjugated secondary antibodies directed against the host species of the primary antibody for 1 h at room temperature. Nuclei were counterstained with bisbenzimide (Hoechst 33342, Thermo Fisher, 62249) (1:1000 in PBS) for 10 min at room temperature. Slides were washed again in PBS, mounted using ProLong Glass Antifade mounting medium (Thermo Fisher, P36980) and protected from light during overnight curing at room temperature. Images were acquired using a Nikon A1R HD25 laser-scanning confocal microscope in the Emory Integrated Cellular Imaging Core with a 60× oil immersion objective (NA 1.4, working distance 0.14 mm).

#### Antibody usage for immunostaining

The primary antibody used for immunostaining was: anti-EpCAM/CD326 (RRID:AB_1089026; BioLegend, 118201; 1:200) at 2.5 μg/ml. Secondary antibodies used for immunostaining were: donkey anti-rat Alexa Fluor 555 (ThermoFisher, A31572; 1:500) and donkey anti-rat Alexa Fluor 647 (ThermoFisher, A21208; 1:500), each at 4 μg/ml.

### Intrahepatic bile duct dissociation and single cell isolation for FACS

Isolation of BECs was adapted from previously described protocols, with minor modifications (Hrncir et al., 2025). Liver lobes were dissected, avoiding the gallbladder and extrahepatic duct to minimize contamination. Tissue was rinsed in Dulbecco's phosphate-buffered saline (DPBS) and finely minced with a razor blade (<0.5 cm^2^ fragments) prior to transferring into 50 ml conical tubes using pre-warmed Liver Wash Buffer (LWB; DMEM-H; Fisher Scientific, 11965118) supplemented with 1% fetal bovine serum (Genesee Scientific, 25-525H), 1% Glutamax (Fisher Scientific, 35050061) and 1% penicillin/streptomycin (Fisher Scientific, 15140122). Tissue fragments were allowed to settle and the supernatant was removed. This wash-sedimentation step was repeated twice with fresh LWB. Samples were pelleted in a swinging bucket centrifuge at 600 ***g*** for 5 min at 4°C and resuspended in RT Liver Digest Buffer [10 ml LWB, 0.15 mg/ml collagenase type XI (Sigma Aldrich, C9407), 0.3 U/ml dispase (Corning, 354235) and 200 µg/ml DNase (Sigma Aldrich, DN25)]. Digestion proceeded for 90 min at 37°C on an end-over-end rotator. Every 30 min, tissue suspensions were pipetted vigorously using a 1 ml pipette for mechanical digestion. After digestion, an equal volume of LWB was added to quench and samples were centrifuged at 200 ***g*** for 5 min at room temperature. Supernatant was decanted and pellets were washed once more with LWB and then centrifuged at 600 ***g*** for 5 min at 4°C. Pellets were resuspended in 5 ml 1× red blood cell (RBC) lysis buffer (BioLegend, 420301) on ice for 5 min (with periodic agitations) then quenched with DPBS and centrifuged at 600 ***g*** for 5 min at 4°C. The supernatant was decanted. For culturing BEC organoids and monolayers, dissociation was stopped and ductal fragments were used.

For single cell isolation, samples were incubated in 3 ml TrypLE (Fisher Scientific, 12605010)+10 μM Y-27632 (Selleck Chemicals, S1049) at 37°C for 12 min with 30 s of vigorous pipetting every 2 min to mechanically dissociate any remaining IHBDs. After 12 min, DPBS was added to quench the TrypLE, and the sample was pelleted at 600 ***g*** for 5 min at 4°C. Cells were resuspended in Sort Media (Advanced DMEM/F12 (ThermoFisher, 12634028) supplemented with 2% B27 supplement without vitamin A (Thermo Fisher, 12587010), 1% N2 supplement (Thermo Fisher, 17502048), 2 mM Glutamax, 10 mM HEPES (Thermo Fisher, 15630080), 100 U/ml penicillin/streptomycin and 0.1% DNase. After resuspension, single cell suspensions were incubated on ice for 1 h with anti-CD31-APC (RRID:AB_312917; BioLegend, 102510; 1:100), anti-CD45-APC (RRID:AB_312977; BioLegend, 103112; 1:100) and anti-CD326/EpCAM-APC/Cy7 (RRID:AB_2098648; BioLegend, 118218; 1:100). After washing in Sort Media and centrifugation, cells were resuspended and stained with 7-AAD and Annexin V to identify non-viable and apoptotic populations, respectively. Cells were analyzed and sorted on a Sony SH800S cell sorter. Nonepithelial and dead cells were excluded by gating out CD31^+^, CD45^+^, 7-AAD^+^ and Annexin V^+^ events. BECs and HybHeps were collected based on EGFP^+^ signal (Fig. S1A).

### Single cell RNA-sequencing

EGFP^+^ cells isolated by FACS were fixed and processed for single cell RNA sequencing following the manufacturer's guidelines for whole cells (Parse Biosciences, SB1001). Libraries were prepared using split-pool barcoding defined by the Evercode WT V2 kit (Parse Biosciences, SB1001). Pooled libraries were sequenced on the Illumina NovaSeq S4 flow cell in one lane (R1: 74 cycles, i7 index: 6 cycles; R2: 86 cycles, i5 index: 0 cycles) in the Emory Nonhuman Primate Genomics Core.

#### scRNA-seq analysis

The Parse Biosciences bioinformatics pipeline (v0.9.6p) was used to align reads to the *Mus musculus* reference genome (RefSeq GRCm39). The resulting filtered feature count matrices were further processed and analyzed using the *Seurat* package (v5.0.1) (Hao et al., 2021). Low-quality cells were filtered out if they contained fewer than 200 unique molecular identifiers (UMIs) or fewer than 100 detected features. Seurat objects for individual samples were merged using the *merge()* function.

Data normalization was performed using Seurat's default LogNormalize method via the *NormalizeData()* function. Highly variable features were identified using *FindVariableFeatures()* and data scaling was carried out using the *ScaleData()* function. Principal component analysis (PCA) was performed with *RunPCA()* and clustering was conducted using the *FindNeighbors()* and *FindClusters()* functions with the first 23 principal components and a clustering resolution of 0.6. The optimal clustering resolution was determined by evaluating the clustering tree generated with the *clustree* package (v0.5.1). Nonlinear dimensionality reduction was performed using uniform manifold approximation and projection (UMAP) via the *RunUMAP()* function implemented in Seurat. Batch correction based on sample identity was performed using Harmony Integration (θ=3) (v1.2.3) (Korsunsky et al., 2019).

Cluster-specific marker genes and genes differentially expressed between conditions were identified through differential gene expression (DGE) analysis using the *FindAllMarkers()* and *FindMarkers()* functions, with the MAST statistical test (Finak et al., 2015) implemented in Seurat (Tables S2-S3, S6-S10).

Single-sample gene set enrichment analysis (ssGSEA) was performed using the *runEscape()* function from the *escape* package (v2.2.3). For differential enrichment analysis, ssGSEA scores were first normalized using the *performNormalization()* function. The normalized scores were then compared across clusters using the *FindAllMarkers()* function with the Wilcoxon rank-sum test. Additionally, gene signature scores were calculated using *UCell* (v2.10.1).

For gene set enrichment analysis (GSEA), the DGE results table was filtered to include only genes expressed in at least 30% of the cells (pct.1>0.3) from the BDL samples. GSEA was performed using the GSEA() function from the clusterProfiler package (v4.14.6).

#### Metagenes

Sham-specific clusters (Sham 1, Sham 2, Sham 3 and Sham 4) were subset and converted to the H5AD file format using the *SeuratDisk* package (v0.0.0.9015). The minimal combinations of marker genes sufficient to distinguish among the Sham clusters were identified using NS-Forest (v3.9) with the following parameters: *n_trees=1000*, *n_top_genes=30*, *n_binary_genes=25*, *n_genes_eval=20* and *beta=0.5*. Metagenes identified by NS-Forest were visualized based on expression or co-expression of the gene(s) using the *VlnPlot()* and *FeaturePlot()* functions.

### Mouse intrahepatic biliary organoid and monolayer culture

#### Whole-duct culture

Following the intrahepatic bile duct dissociation, ductal fragments were resuspended in 500 μl1 ml Advanced DMEM/F12 (Gibco) based on pellet size. To assess the density for plating, a 10 μl aliquot was examined by light microscopy and the sample was further diluted if necessary, in Advanced DMEM/F12 (Gibco). The ductal prep was mixed with Cultrex Type II Growth Factor Reduced extracellular matrix (R&D Biosystems, 3533-010-02) to a final concentration of 70% Cultrex brought to a total volume sufficient to plate 30 μl droplets in a pre-warmed 48-well plate. The cultrex-duct droplets were allowed to polymerize at 37°C for 25 min. After polymerization, droplets were covered with 200 μl of Biliary Isolation Media (50% Advanced DMEM/F12, 40% WNT3A-conditioned media, 10% RSPO1-conditioned media, 2% B27 supplement without vitamin A, 1% N2 supplement, 2 mM Glutamax, 10 mM HEPES, 100 U/ml penicillin/streptomycin, 50 ng/ml recombinant murine EGF (Thermo Fisher, PMG8041), 100 ng/ml recombinant human Noggin (VWR International, 120-10C), 100 ng/ml recombinant human FGF10 (Peprotech, 100-26), 10 nM recombinant human gastrin (Sigma, G9145), 5 ng/ml recombinant human HGF (PeproTech, 100-39H), 10 mM nicotinamide (Sigma Aldrich, N0636-100G), 10 μM forskolin (Selleck Chemicals, S2449) and 10 μM Y-27632. After 48 h in Biliary Isolation Media, media were replaced every other day with Biliary Expansion Media (WNT3A-conditioned medium was replaced with Advanced DMEM/F12, and Noggin and Y-27632 were removed).

Organoids were cultured for 7 days between passages. For passaging, 1 ml of 1% BSA in PBS was added to each Cultrex-duct droplet. Mechanical dissociation by pipetting resuspended the droplet, scraping the bottom of the well for full recovery. Organoids were pelleted in a swinging bucket centrifuge at 600 ***g*** for 5 min at 4°C. Supernatant and Cultrex layer were pipetted off carefully to avoid disturbing the organoid pellet. The pellet was resuspended in 1 ml TrypLE supplemented with 10 μM Y-27632 and incubated at 37°C for 4 min. TrypLE was quenched using 1 ml of 1% BSA in PBS and organoids were further dissociated by pipetting 25 times. The sample was pelleted at 600 ***g*** for 5 min at 4°C. Organoid fragments were replated at a 1:2 ratio as 30 μl droplets (70% Cultrex: 30% Advanced DMEM/F12) in pre-warmed 48-well plates, as described above. After each passage, BECs were cultured initially in Biliary Isolation Media and switched to Biliary Expansion Media after the first media change 48 h later.

#### BEC monolayer culture

Following the second passage, BEC organoid fragments were passaged into chamber slides coated with 10% Cultrex. BECs were initially grown in Biliary Isolation Media and switched to BEC Expansion Media after the first media change 48 h later. Monolayers were expanded until confluent (7d-10d).

### 7-plex HCR FISH and immunofluorescence

HCR FISH was performed on both tissue sections and cultured BEC monolayers from wild-type mice (Sox9^EGFP−^) following the manufacturer's protocol for fixed-frozen tissue sections with some modification. For BEC monolayers, after medium was removed from each well and the chamber device removed, samples were treated identically to fixed-frozen tissue.

#### HCR FISH

Samples were rehydrated with PBS for at least 3 min at room temperature and fixed by adding slides to slide mailers with 4% PFA made from 16% paraformaldehyde stocks (PFA; Thermo Fisher Scientific, 043368-9M) for 10 min on ice. Slides were transferred into a slide mailer containing 50% ethanol for 5 min at room temperature. This was followed by sequential washes in 70% ethanol and then two washes in 100% ethanol. Slides were quickly rinsed with DPBS and extra liquid was removed using a Kimwipe. Slides were transferred to a prewarmed (37°C) humidifier slide box. Prewarmed (37°C) HCR Hybridization Buffer (Molecular Instruments, v3.0) was added to each slide and incubated for 15 min at 37°C. Slides were then tapped on the slide of the box to remove the buffer and probes were added to prewarmed (37°C) Hybridization Buffer at either 16 nM (*Palld*, *Stat5b*, *Pde4c* and *Fos*) or at 8 nM (*Sema3e*, *Fam107b* and *Tspan8*). Slides were covered with parafilm to prevent evaporation, molecular-grade water was added to the humidifier box and slides were incubated for 16-18 h at 37°C. Parafilm was removed from each slide and excess buffer was carefully tapped off. Slides were quickly rinsed with prewarmed (37°C) HCR Probe Wash Buffer (Molecular Instruments, v3.0) and then transferred to a slide mailer. Prewarmed Probe Wash buffer was diluted with saline-sodium citrate (SSC) (Invitrogen, 15557036) and Tween (Thermo Fisher Scientific, 85113) buffer (75%:25%, 50%:50%, 25%:75%, 0%:100%), and slides were incubated at each dilution in slide mailers for 15 min at 37°C. Slides were then incubated with fresh 100% SSC-Tween for 5 min at room temperature followed by a quick DPBS rinse. Excess liquid was dried with a Kimwipe and slides were transferred to a slide box. HCR Amplification Buffer (Molecular Instruments, v3.0) was prewarmed (37°C) for resuspension before adding to each slide, and incubated for 30 min at room temperature. Hairpins were snap-cooled (3 min incubation at 95°C followed by 30 min at room temperature in a dark drawer) and added to HCR Amplification Buffer at 60 nM. Slides were tapped to remove excess liquid and the hairpins were diluted in HCR Amplification Buffer added to each slide. Parafilm was added to prevent evaporation and slides were incubated 16-18 h at room temperature. Parafilm was removed, and slides were incubated in SSC-Tween for 5 min at room temperature. Slides were then transferred to a slide mailer and incubated in fresh SSC-Tween for 30 min at room temperature; this step was repeated. Slides were then transferred back to a slide box for immunofluorescence.

#### Immunofluorescence

Tissues were washed three times for 5 min each in PBS in slide mailer. Slides were transferred to a staining box and tissues were incubated in 5% normal donkey serum for 45 min. Primary antibody diluted in PBS was applied overnight at 4°C. The following day, primary antibody solution was discarded, and slides were rinsed three times for 5 min each with PBS. Secondary antibody raised against host species of the primary antibody was incubated in PBS overnight at 4°C. The following day, Nuclei were counterstained with bisbenzimide (1:1000 in PBS) for 10 min at room temperature. Slides were then washed three times for 5 min each with PBS, mounted with ProLong Glass Antifade and protected from light during overnight curing at room temperature.

### 9-color image acquisition on a confocal microscope

Tissue samples and fixed monolayers were imaged on the Leica STELLARIS 8 in the Emory Integrated Cellular Imaging Core. Each laser (excitation, ex), emission range (em) and detector group are as follows: bisbenzimide (405 ex, 425-450 em, HyDS1), *Palld*-488 (488 ex, 495-505 em, HyDX1), *Stat5b*-514 (520 ex, 540-555 em, HyDS2), *Pde4c-*546 (560 ex, 575-585 em, HyDS1), *Sema3e*-594 (594 ex, 605-625 em, HyDS3), EpCAM-647 (653 ex, 660-6-85 em, HyDS3), *Fos*-700 (700 ex, 705-740 em, HyDX2), *Fam107b*-750 (750 ex, 755-770 em, HyDX1) and *Tspan8*-790 (790 ex, 800-850 em, HyDX2). Channels were acquired in pairs (except 405) simultaneously as listed: bisbenzimide; *Palld* and EpCAM; Stat5b and Fos); Pde4c and Fam107b; and Sema3e and Tspan8. Each pair was acquired as an entire frame before switching to the next pair. All image fields from tissue samples were acquired using the 63× oil objective (HC PL APO 63×/1.40 NA oil CS2) using a range of 1.49-5.38× zoom and a *z*-step no larger than 0.3 μm. All image fields from fixed monolayers were acquired using the 40× oil objective (HC PL APO 40×/1.3 NA oil, PH3 CS2) with a *z*-step no larger than 0.3 μm.

### 9-color image quantification and data analysis

Tissue samples and fixed monolayers were quantified using the Leica Application Suite (LAS) X (RRID:SCR_013673). Maximum Intensity Projection was performed on each *z*-stack image. For tissue sections, segmentation of BECs was based on positive EpCAM signal, resulting in a region of interest (ROI) for each BEC imaged. Because the EpCAM staining failed on BEC monolayers, segmentation of BECs was based on autofluorescence in the 647 channel. Mean intensity was calculated in the LAS X software for each individual channel (Tables S5, S11).

#### Metagene scoring

The mean intensity values for each channel corresponding to the 7 metagenes (488-*Palld*, 514-*Stat5b*, 546-*Pde4c*, 594-*Sema3e*, 700-*Fos*, 750-*Fam107b* and 790-*Tspan8*) was normalized by z-scoring in both tissue sections and monolayers. For tissue sections, z-scoring was calculated across all fields within each biological replicate (Table S5). For monolayers, z-scoring was calculated within each field for each biological replicate (Table S11). Using the z-scores, a metagene score was calculated for each cell based on the average z-score of the gene(s) composing the metagene (Tables S5, S11). The identity for each BEC was determined by the highest metagene score (Tables S5, S11).

#### Duct diameter

The duct diameter was calculated by averaging the length of two sets of perpendicular lines (Fig. S5). Each line was drawn across the duct lumen, from the basolateral membranes of opposing BECs.

## Supplementary Material



10.1242/develop.205690_sup1 Supplementary information

Table S1. Gene sets for UCell analysis in Supplementary Figure 2.

Table S2. Differential expression analysis for Sham 1-6 and BDL 1-7 clusters.

Table S3. Differential expression analysis for Sham 1-4 clusters.

Table S4. Ranked Δpct from differential expression analysis for Sham 1-4 clusters

Table S5. HCR FISH-derived metagene scores for BECs in tissue sections.

Table S6. Differential expression analysis for BDL 3 vs BDL 4.

Table S7. Differential expression analysis for BDL 1 vs Sham 1.

Table S8. Differential expression analysis for BDL 2 vs Sham 2.

Table S9. Differential expression analysis for BDL 3 vs Sham 3.

Table S10. Differential expression analysis for BDL 4 vs Sham 4.

Table S11. HCR FISH-derived metagene scores for BECs in monolayers.
