# Supplementary material for: Multi-gene biomarkers reveal spatial organization and subpopulation-specific damage response in biliary epithelial cells
Source: Development. 2026 Oct 12;153(19):dev205690. doi: 10.1242/dev.205690 (PMC13723405; doi:10.1242/dev.205690)
Supplement: Supplementary file 1 [file develop-153-205690-s1.pdf]

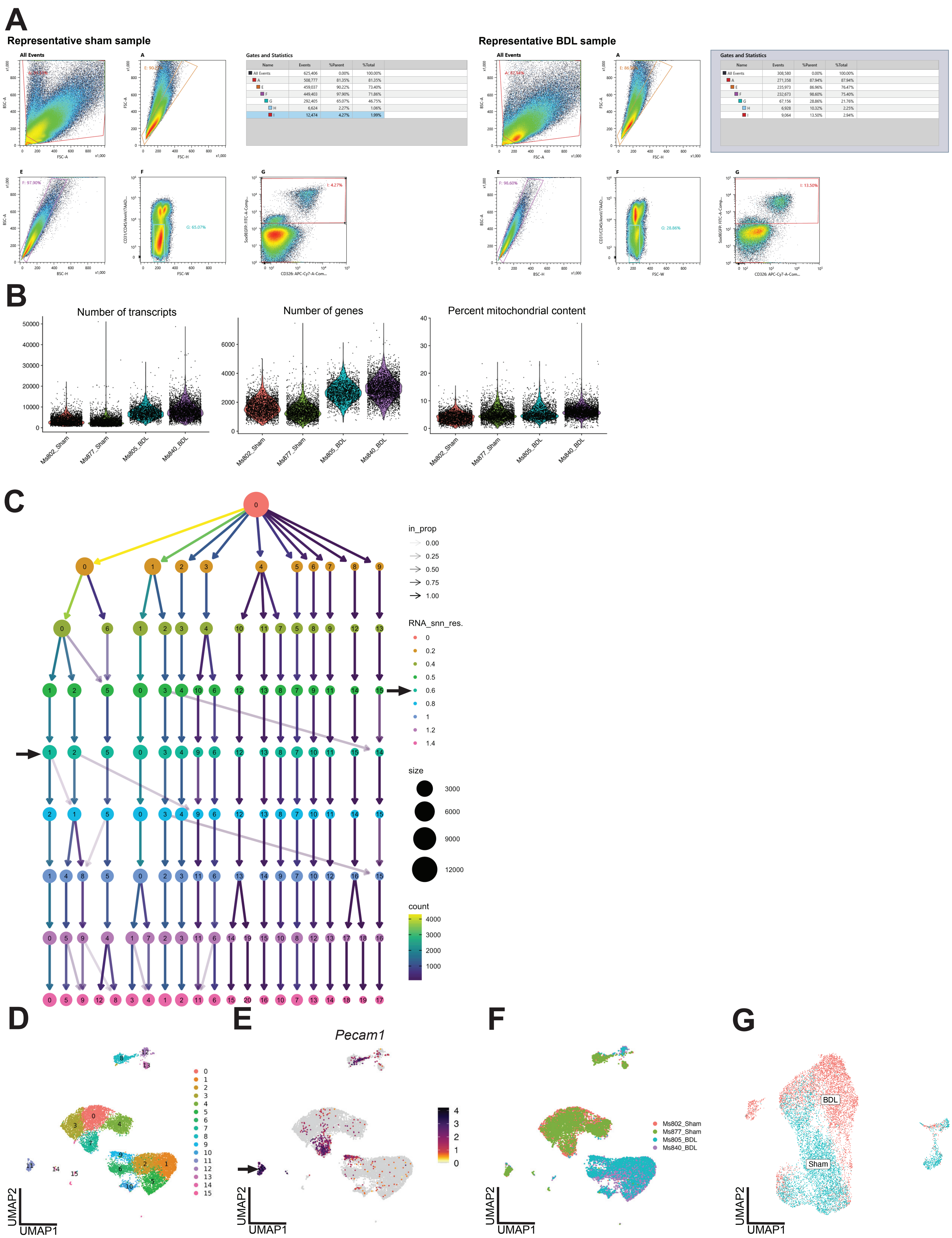
A
Representative sham sample
Representative BDL sample
All Events
Gates and Statistics
B
Number of transcripts
Number of genes
Percent mitochondrial content
Ms802_Sham
Ms877_Sham
Ms805_BDL
Ms840_BDL
C
in_prop
0.00
0.25
0.50
0.75
1.00
RNA_snn_res.
0
0.2
0.4
0.5
0.6
0.8
1
1.2
1.4
size
3000
6000
9000
12000
count
4000
3000
2000
1000
D
E
Pecam1
F
G
BDL
Sham
UMAP1
UMAP2

Fig. S1. Transcriptomic analysis of Sox9EGFP+ cells in sham and BDL. (A) Strategy for FACS isolation of $Sox9^{EGFP+}$ cells from mouse liver tissue. (B) Final QC metrics show downstream analyses on cells with ≥ 1,000 transcripts detected and with mitochondrial RNA content ≤ 50%. (C) Visualization of clustree output, with nine different resolutions based on unsupervised clustering. Black arrow indicates the selected resolution for downstream analysis (res = 0.6; 16 total clusters). (D) Representative UMAP of the 16 clusters, labeled to correspond to the numbers in Fig. 1D. (E) Exclusion of cluster 11 is based on *Pecam1* expression, which marks contaminating endothelial cells. (F) Samples cluster based on experimental condition, suggesting that gene expression changes resulting from cholestasis drive the major differences between clusters (sham: n=2 mice and BDL: n= 2 mice). (G) Representative UMAP following Harmony reveals persistent separation between sham and BDL samples, even after stringent integration.

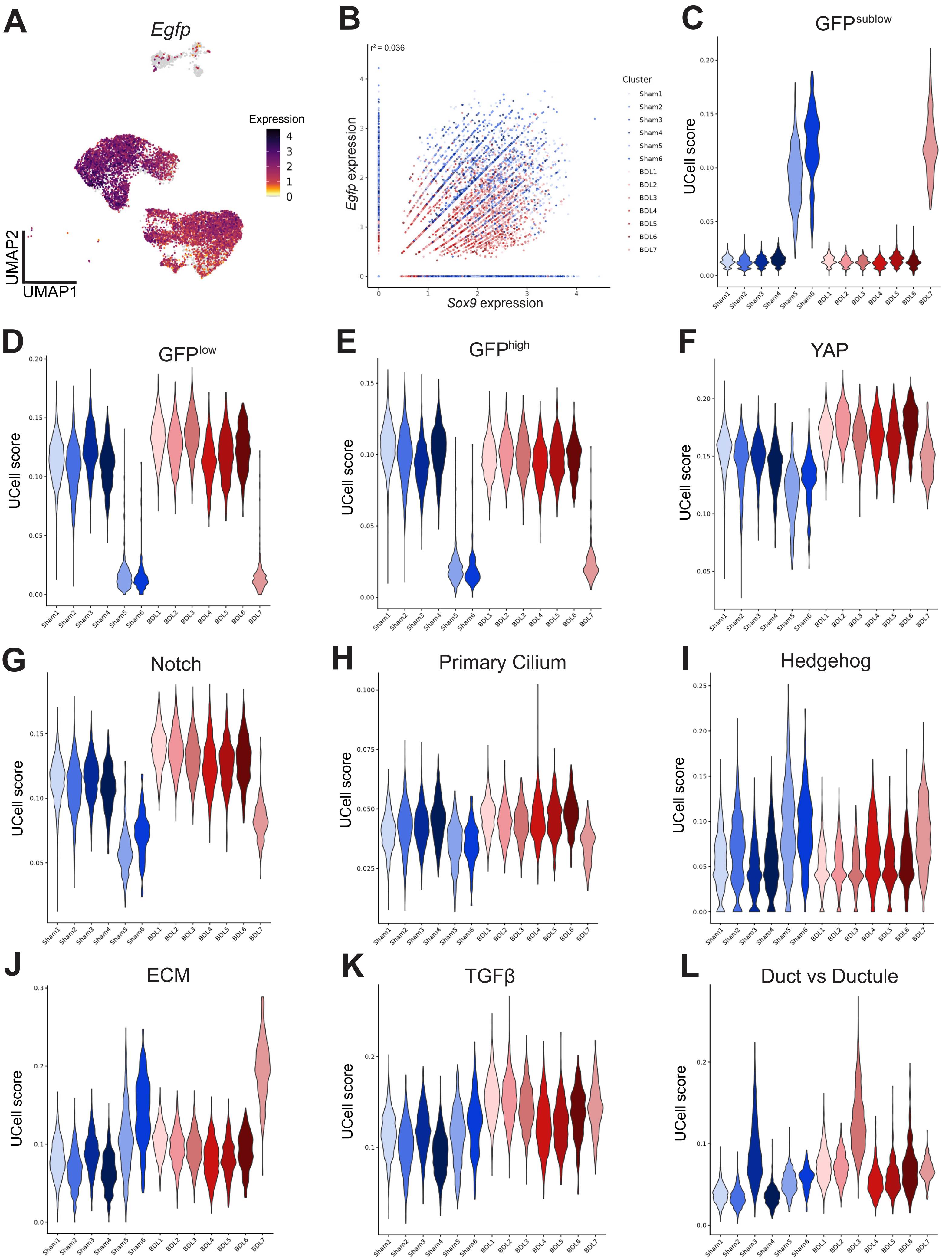
A
Egfp
Expression
4
3
2
1
0
UMAP2
UMAP1
B
r² = 0.036
Egfp expression
Sox9 expression
Cluster
Sham1
Sham2
Sham3
Sham4
Sham5
Sham6
BDL1
BDL2
BDL3
BDL4
BDL5
BDL6
BDL7
C
GFPsublow
UCell score
D
GFPlow
UCell score
E
GFPhigh
UCell score
F
YAP
UCell score
G
Notch
UCell score
H
Primary Cilium
UCell score
I
Hedgehog
UCell score
J
ECM
UCell score
K
TGFβ
UCell score
L
Duct vs Ductule
UCell score
Sham1
Sham2
Sham3
Sham4
Sham5
Sham6
BDL1
BDL2
BDL3
BDL4
BDL5
BDL6
BDL7

Fig. S2. Gene signature scoring identifies BEC and HybHep clusters and reflects limited variability between BEC clusters. (A) UMAP visualization of *Egfp* expression across all clusters. *Egfp* is enriched in Sham 1–4 and BDL 1–5 clusters, consistent with BEC identity, and reduced in Sham 5–6 and BDL 6 clusters, consistent with HybHep populations. (B) Relationship between *Sox9* transcripts and *Egfp* transcripts reveals no correlation to specific BEC subpopulations. (C–E) Violin plots showing UCell enrichment scores for transcriptomic signatures derived from previously published bulk RNA-seq of $Sox9^{EGFP}$ populations. Gene signatures associated with GFP^{high} and GFP^{low} populations, which correspond to BECs, are enriched in Sham 1–4 and BDL 1–5 clusters. GFP^{sub} signature associated with HybHeps is enriched in Sham 5–6 and BDL 6 clusters. (F–K) Violin plots showing enrichment of published gene sets associated with signaling pathways and cellular features previously studied in BEC biology, including YAP, Notch, primary cilium, Hedgehog, extracellular matrix (ECM), and TGF-β signaling. These pathways show broad expression across BEC clusters with variable enrichment patterns between Sham and BDL conditions. Notably, Hedgeog signaling and ECM signatures are enriched in Sham 5–6 and BDL 6 clusters, suggesting a potential role in HybHeps. (L) Violin plot showing enrichment of a previously defined duct versus ductule gene signature in Sham 3 and BDL 3, suggesting these two clusters may be localized to large ducts. Simple linear regression (B).

Development • Supplementary information

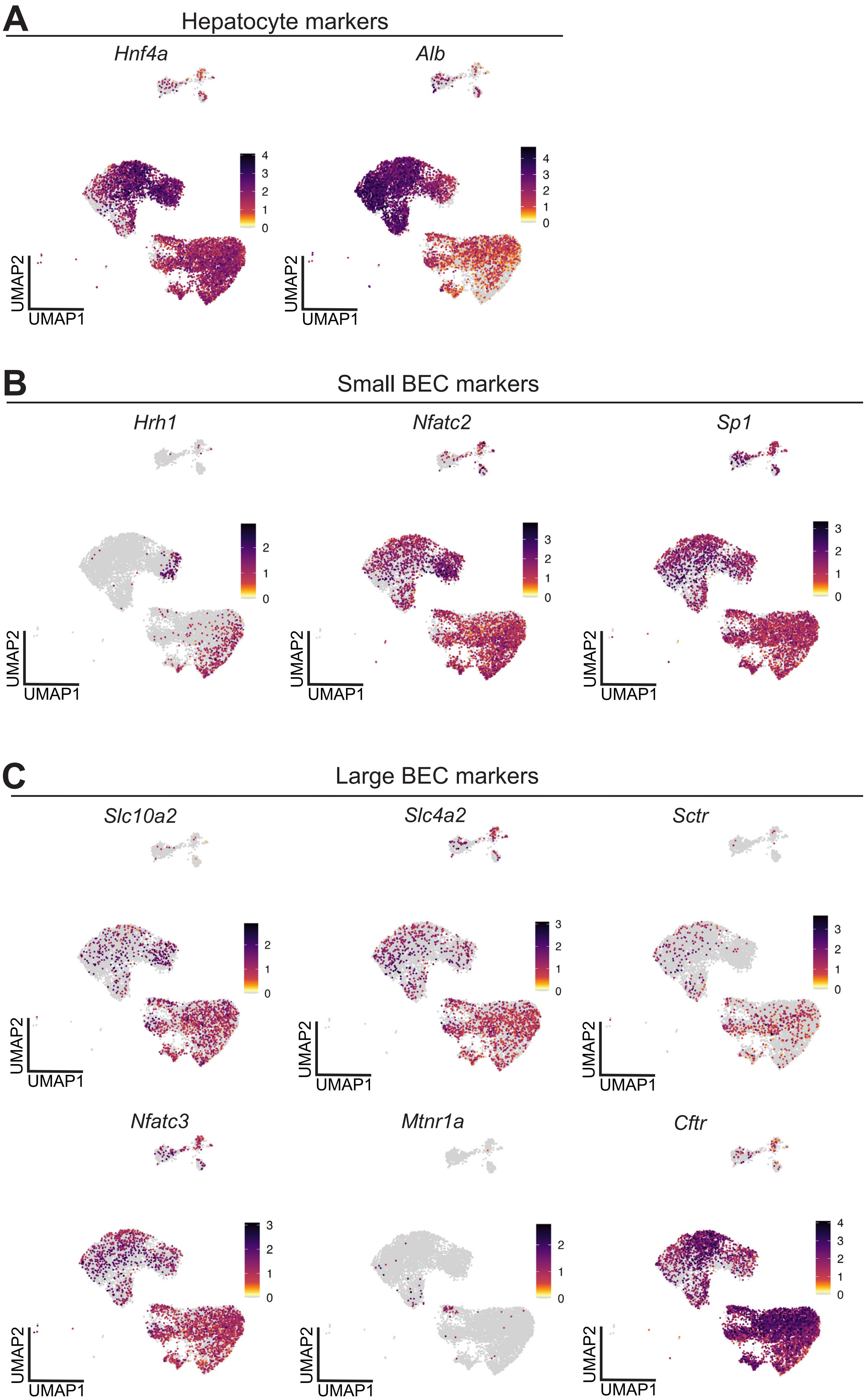

Development • Supplementary information

Fig. S3. Canonical hepatocyte and BEC markers are broadly expressed across BEC clusters. (A) Expression of canonical hepatocyte markers (*Hnf4a* and *Alb*) are detectable across multiple BEC clusters, with variable expression levels and no clear cluster-specific pattern. (B) Expression of reported small ductule–associated markers (*Hrh1*, *Nfatc2*, and *Sp1*) across clusters. *Hrh1* shows localized enrichment to a subpopulation of BECs within Sham 3 in Sham 3 and BDL 3 and 4. The remaining markers are broadly expressed among BEC clusters. (C) Expression of reported large bile duct–associated markers (*Slc10a2*, *Slc4a2*, *Sctr*, *Nfatc3*, *Mtnr1a*, and *Cftr*). Most markers exhibit widespread expression across BEC clusters.

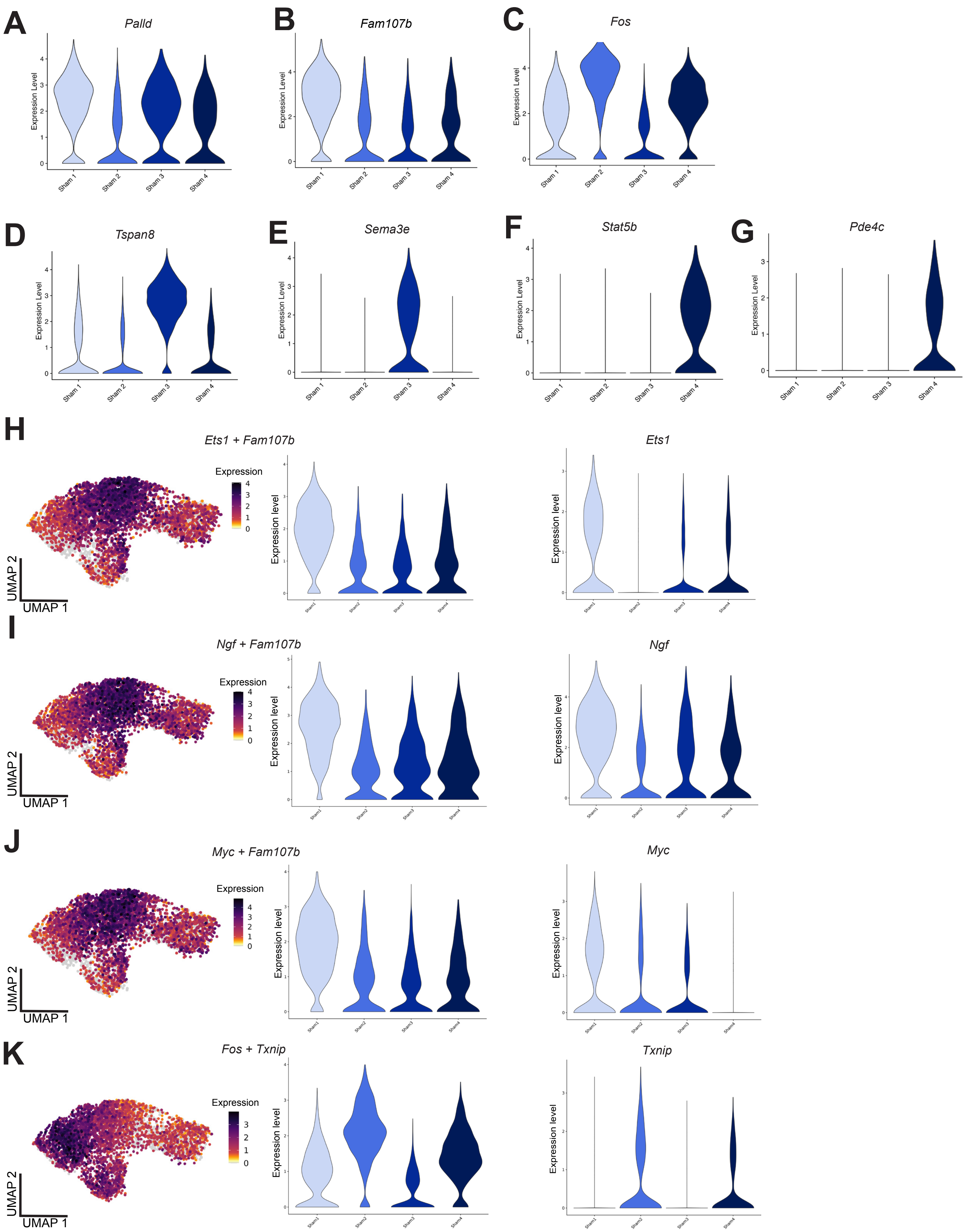
A
Palld
B
Fam107b
C
Fos
D
Tspan8
E
Sema3e
F
Stat5b
G
Pde4c
Expression Level
Sham 1
Sham 2
Sham 3
Sham 4
H
Ets1 + Fam107b
Ets1
I
Ngf + Fam107b
Ngf
J
Myc + Fam107b
Myc
K
Fos + Txnip
Txnip
Expression
Expression level
UMAP 1
UMAP 2
Sham1
Sham2
Sham3
Sham4

Fig. S4. NSForest identifies minimal gene sets with binary expression defining four BEC subpopulations in sham. Across sham BEC clusters, each metagene shows strong enrichment in a single cluster and minimal expression in the remaining sham clusters, achieving binary expression. (A–B) Expression of Palld and Fam107b (Metagene 1) defines the Sham 1 subpopulation. (C) Expression of Fos (Metagene 2) uniquely defines Sham 2. (D–E) Expression of Tspan8 and Sema3e (Metagene 3) defines the Sham 3 subpopulation. (F–G) Expression of Stat5b and Pde4c (Metagene 4) defines the Sham 4 subpopulation. (H–J) Ets1, Ngf, and Myc as alternative genes to Palld in Metagene 1, each co-expressed with Fam107b. Single-gene expression plots are shown for each alternative. (K) Txnip co-expressed with Fos as an alternative Metagene 2. Single-gene expression plot is shown for Txnip.

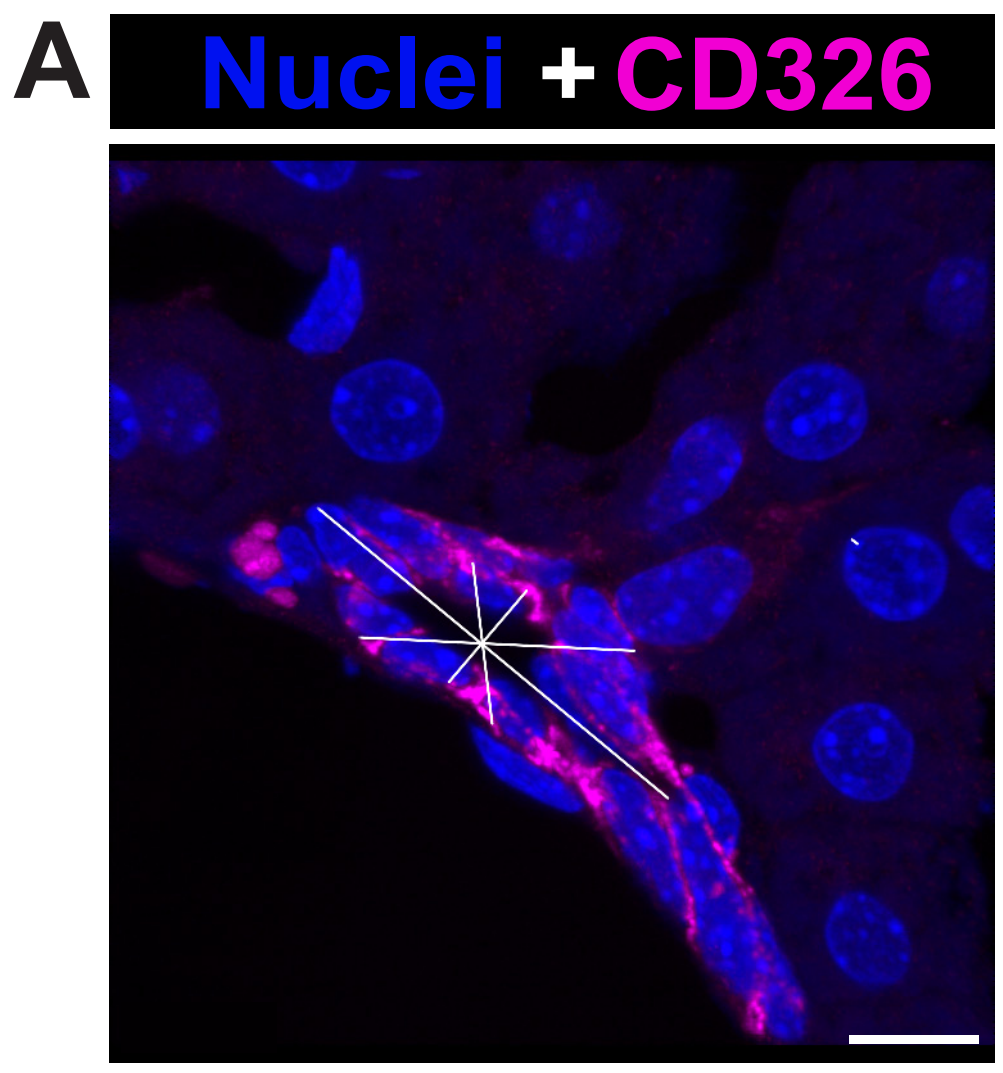

B

Line 1	33.72 µm
Line 2	10.71 µm
Line 3	22.34 µm
Line 4	11.22 µm
Average	19.50 µm

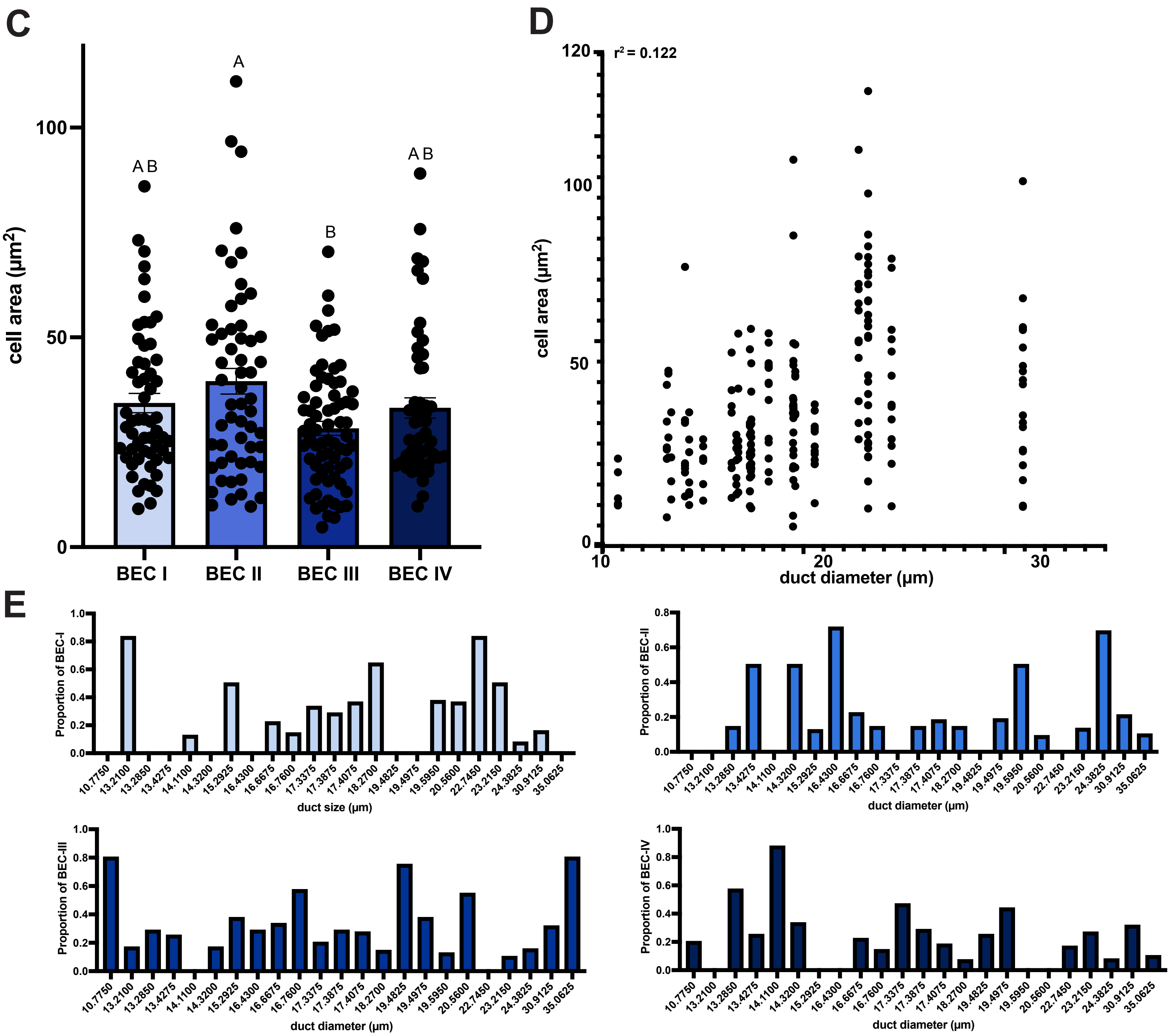

Fig. S5. ***In situ* analysis of metagenes relative to duct diameter.** (A) Representative imaging field for duct quantification. Two sets of perpendicular lines were drawn across the ductal lumen from the basolateral membranes of opposing BECs to calculate duct diameter. (B) Representative calculation for duct diameter (µm): the average of the length of the 4 lines drawn in Fig. S5A. (C) BEC area (µm^2) is heterogeneous *in vivo* (7.4-110 µm^2), but size does not correlate to specific BEC subpopulations (n = 243 BECs from tissue sections). (D) BEC area and IHBD size (as determined by duct diameter) do not correlate, suggesting BEC size is independent of IHBD size. (E) Quantification of BEC subpopulation composition across all IHBDs imaged by HCR-FISH. Each bar represents the proportion of a given BEC subpopulation within a single duct. one-way ANOVA (C), Data are mean±s.e.m. simple linear regression (D); Scale bar: 10µm (A).

Development • Supplementary information

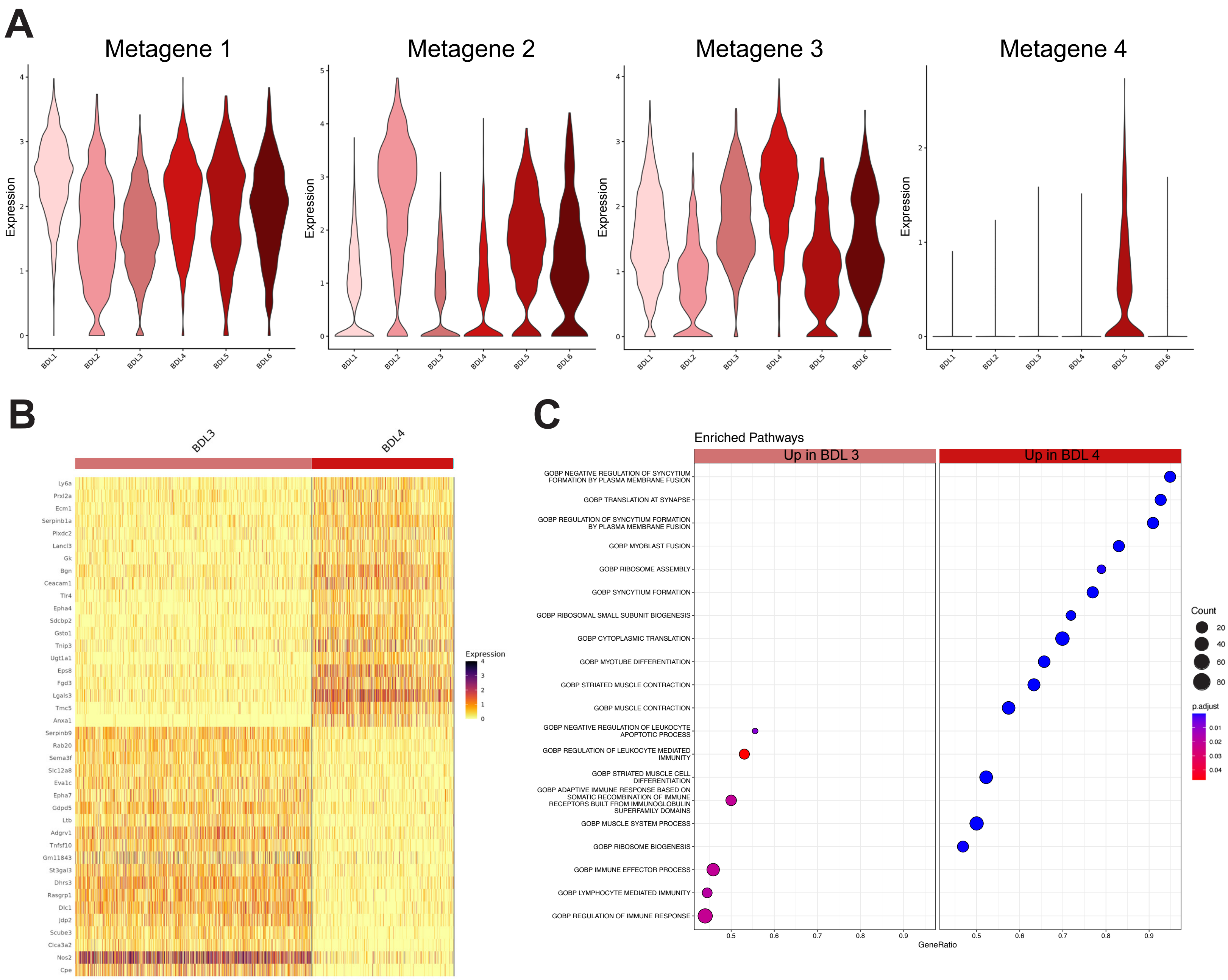

Fig. S6. Metagene enrichment matches BDL to Sham BEC subpopulations. (A) Metagenes 1, 2, and 4 are each enriched in a single BDL subpopulation, while Metagene 3 is enriched in both BDL 3 and BDL 4. (B) Representative results demonstrating the top 20 differentially expressed genes between BDL 3 and BDL 4. (C) GSEA reveals differential enrichment of functional pathways between BDL 3 and BDL 4.

Development • Supplementary information

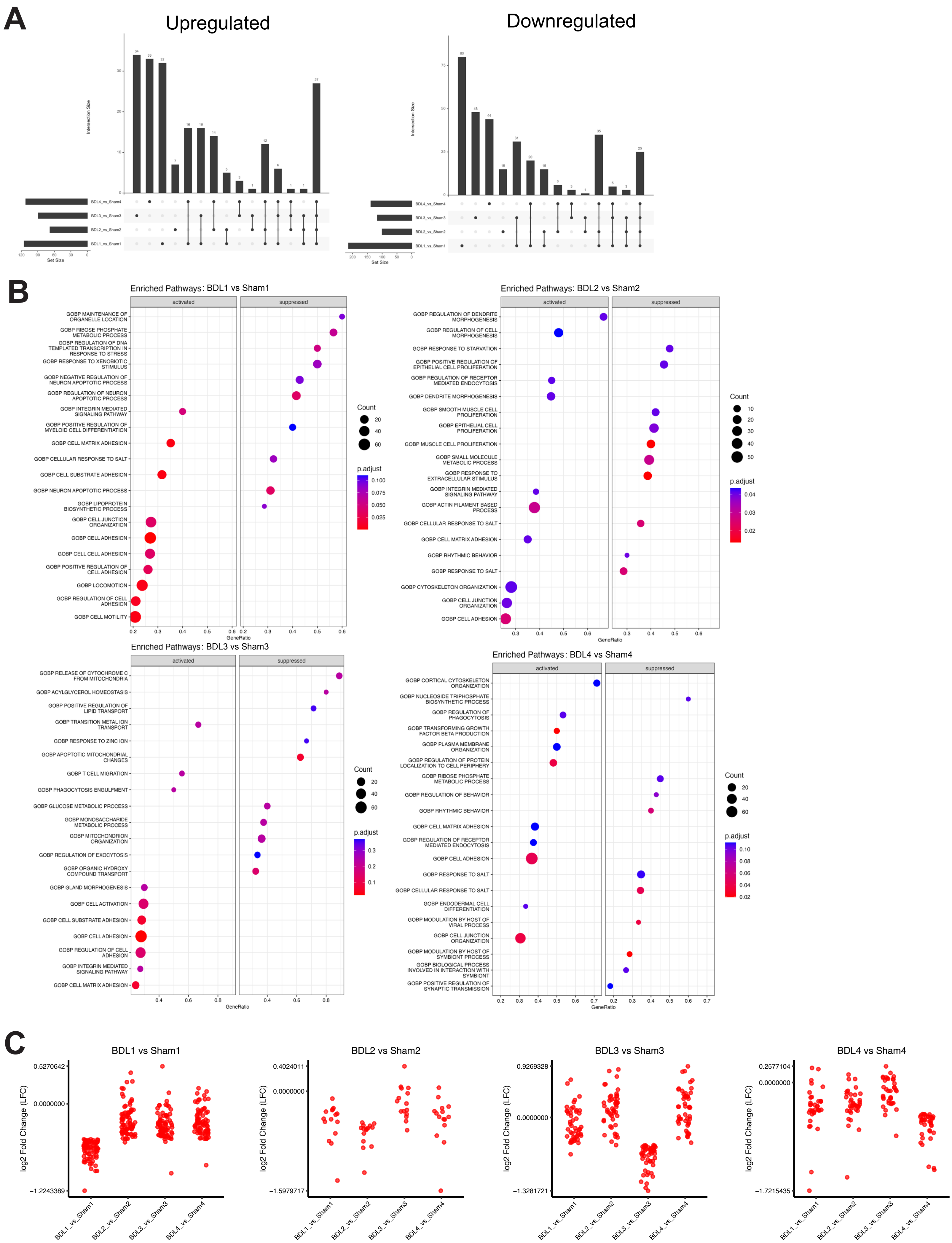

Development • Supplementary information

Fig. S7. Pairwise comparisons between experimental conditions reveals injury-induced transcriptional programs. (A) Differential gene expression analysis identifies a combination of shared and subpopulation-specific DEGs in response to BDL. (B) GSEA showing activated and suppressed pathways in response to injury in each BEC subpopulations. (C) Downregulated gene sets identified from each pairwise comparison are unique to their respective BDL BEC cluster (BDL 1–4).

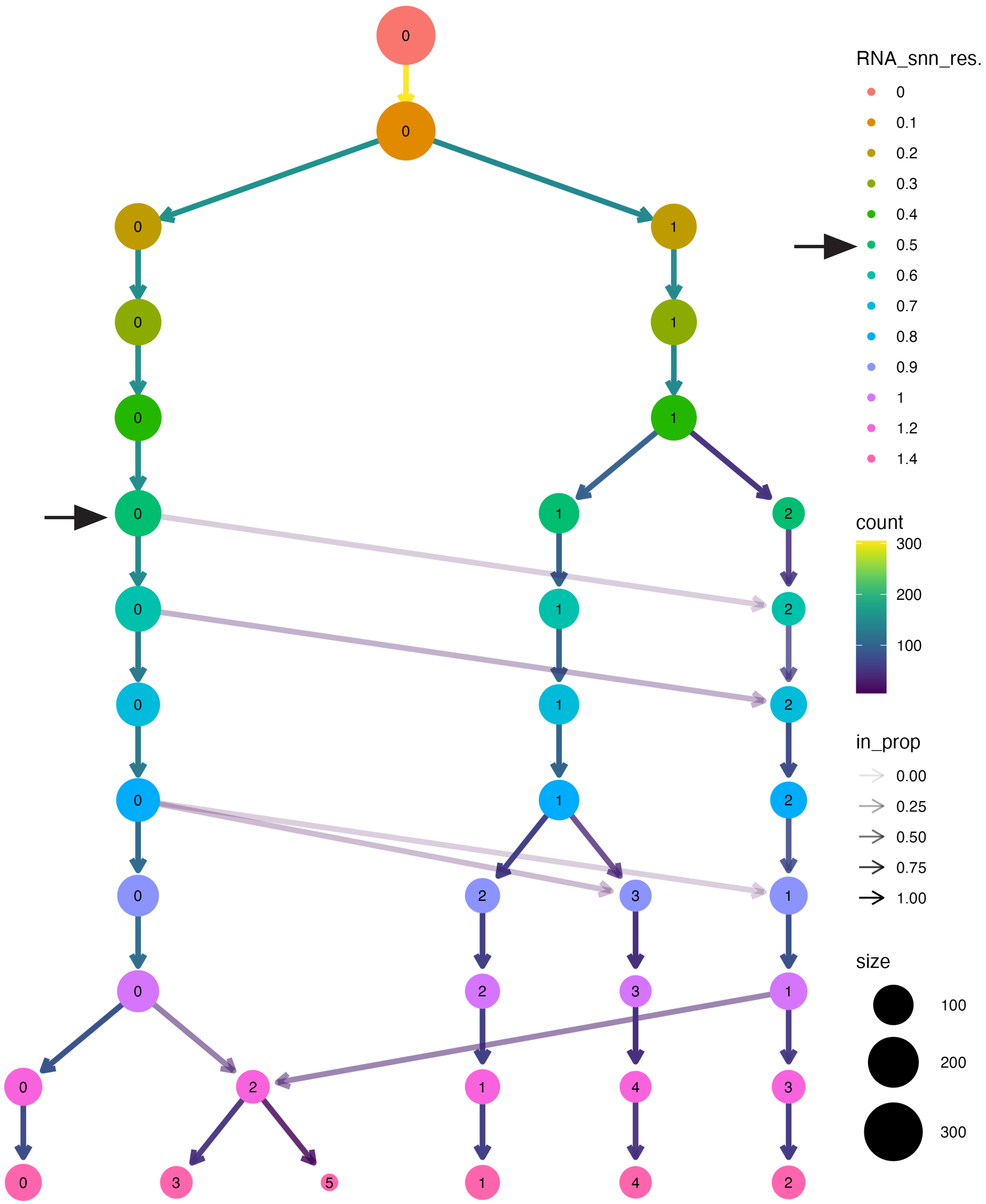

Fig. S8. **Clustree identifies three stable subclusters in the BDL 5 cluster.**

Black arrow indicates cluster resolution selected for analysis.

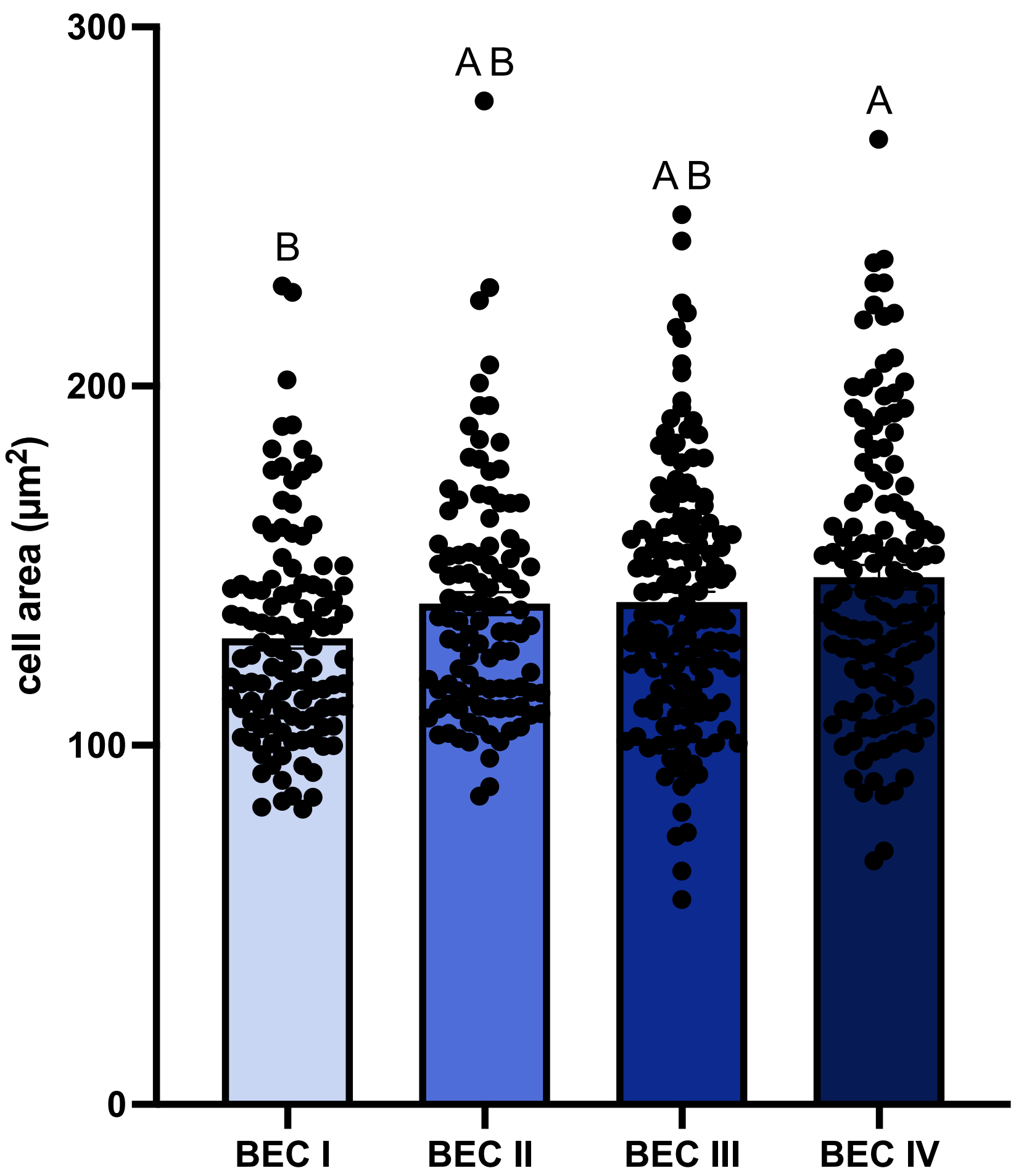

Fig. S9. Metagene-defined BEC subpopulations are not associated with differences in BEC size. BEC area is heterogeneous *in vitro* (57.1-279.4 μm^2), but size does not correlate to specific BEC subpopulations (n = 1,578 BECs from monolayer cultures). one-way ANOVA (C), Data are mean±s.e.m.

Table S1. Gene sets for UCell analysis in Supplementary Figure 2.

Available for download at
https://journals.biologists.com/dev/article-lookup/doi/10.1242/dev.205690#supplementary-data

Table S2. Differential expression analysis for Sham 1-6 and BDL 1-7 clusters.

Available for download at
https://journals.biologists.com/dev/article-lookup/doi/10.1242/dev.205690#supplementary-data

Table S3. Differential expression analysis for Sham 1-4 clusters.

Available for download at
https://journals.biologists.com/dev/article-lookup/doi/10.1242/dev.205690#supplementary-data

Table S4. Ranked ∆pct from differential expression analysis for Sham 1-4 clusters

Available for download at
https://journals.biologists.com/dev/article-lookup/doi/10.1242/dev.205690#supplementary-data

Table S5. HCR FISH-derived metagene scores for BECs in tissue sections.

Available for download at
https://journals.biologists.com/dev/article-lookup/doi/10.1242/dev.205690#supplementary-data

Table S6. Differential expression analysis for BDL 3 vs BDL 4.

Available for download at
https://journals.biologists.com/dev/article-lookup/doi/10.1242/dev.205690#supplementary-data

Table S7. Differential expression analysis for BDL 1 vs Sham 1.

Available for download at
https://journals.biologists.com/dev/article-lookup/doi/10.1242/dev.205690#supplementary-data

Table S8. Differential expression analysis for BDL 2 vs Sham 2.

Available for download at
https://journals.biologists.com/dev/article-lookup/doi/10.1242/dev.205690#supplementary-data

Table S9. Differential expression analysis for BDL 3 vs Sham 3.

Available for download at
https://journals.biologists.com/dev/article-lookup/doi/10.1242/dev.205690#supplementary-data

Table S10. Differential expression analysis for BDL 4 vs Sham 4.

Available for download at
https://journals.biologists.com/dev/article-lookup/doi/10.1242/dev.205690#supplementary-data

Table S11. HCR FISH-derived metagene scores for BECs in monolayers.

Available for download at
https://journals.biologists.com/dev/article-lookup/doi/10.1242/dev.205690#supplementary-data
