# Supplementary material for: Multi-gene biomarkers reveal spatial organization and subpopulation-specific damage response in biliary epithelial cells
Source: Development. 2026 Oct 12;153(19):dev205690. doi: 10.1242/dev.205690 (PMC13723405; doi:10.1242/dev.205690)
Supplement: Supplementary file 2 [file develop-153-205690_review_history.pdf]

Development

Multi-gene biomarkers reveal spatial organization and subpopulation-specific damage response in biliary epithelial cells

Kendall G. Kanakanui, Fransky Hantelys, Hannah R. Hrncir, Sergei Bombin and Adam D. Gracz
DOI: 10.1242/dev.205690

Editor: James M Wells

Review timeline
Original submission: 24 March 2026
Editorial decision: 29 April 2026
First revision received: 20 August 2026
Accepted: 15 September 2026

Original submission

First decision letter

MS ID#: dev.205690

MS TITLE: Multi-gene biomarkers reveal spatial organization and subpopulation-specific damage response in intrahepatic cholangiocytes

AUTHORS: Kendall Kanakanui; Fransky Hantelys; Hannah Hrncir; Sergei Bombin; Adam D Gracz

Dear Dr Gracz,

I have now received all the referees' reports on the above manuscript, and have reached a decision. The referees' comments are appended below.

As you will see, the referees express considerable interest in your work, but have some significant criticisms and recommend a substantial revision of your manuscript before we can consider publication. If you are able to revise the manuscript along the lines suggested, which may involve further experiments, I will be happy to receive a revised version of the manuscript. Your revised paper will be re-reviewed by one or more of the original referees, and acceptance of your manuscript will depend on your addressing satisfactorily the reviewers' major concerns. Please also note that Development will normally permit only one round of major revision. If it would be helpful, you are welcome to contact us to discuss your revision in greater detail. Please send us a point-by-point response indicating your plans for addressing the referees' comments, and we will look over this and provide further guidance.

Please attend to all of the reviewers' comments and ensure that you upload both a 'clean' version of your Word file, along with a highlighted version clearly showing where you have made changes in the revised manuscript. Please avoid using 'Tracked changes' in Word files as these are lost in PDF conversion. I should be grateful if you would also provide a point-by-point response detailing how you have dealt with the points raised by the reviewers in the 'Response to Reviewers' box. If you do not agree with any of their criticisms or suggestions please explain clearly why this is so.

Reviewer 1

In manuscript dev.205690, Kanakanui and colleagues set out to identify biliary epithelial cell (BEC) subpopulations during homeostasis and after liver injury and biomarkers that can distinguish those

subpopulations. Previous work by the authors and others has established heterogeneity in BECs (for example, based on the expression level of Sox9). However, in scRNA-seq datasets, BECs are almost always grouped in a single cluster. This suggests that the transcriptional difference among BEC subpopulations is not sufficient to lead to the identification of distinct BEC clusters when analyzed in the context of the whole liver. Given the involvement of BECs in the pathophysiology of a host of liver diseases, it is important to be able to unambiguously identify BECs subpopulations and their distribution in control and disease livers, highlighting the value of identifying makers that can reliably distinguish them.

To address this unmet need, the authors took advantage of a Sox9-EGFP alleles from their previous work to isolate Sox9+ cells from adult mouse livers, with or without bile duct ligation (BDL) as a form of cholestatic liver injury and subjected the isolated cells to scRNA-seq. The authors found that BECs from control and BDL livers fall into distinct clusters. Analysis of known hepatobiliary markers and DEGs along with GSEA showed that the control and BDL clusters are indeed transcriptionally distinct but failed to identify a manageable number of specific biomarkers that exhibit a binary expression for each cluster. Therefore, the authors applied a machine learning algorithm called NS-Forest and identified minimal biomarker sets composed of 1-2 genes for each cluster (termed metagenes). Using a 7-panel HCR-FISH covering all the markers in the above-mentioned metagenes, the authors analyzed the spatial distribution of the four BEC subpopulations in control livers and found that 1) Each duct contains at least two different BEC subpopulations, 2) Some BECs preferentially populate ducts of a certain size, and 3) BEC subpopulations tend to form neighborhoods. Using the metagenes, the authors reassigned BEC clustering in the BDL liver and identified those BDL clusters that are capable of entering the cell cycle upon cholestatic injury. Finally, using in vitro cultures of primary BECs established from wild-type bile duct fragments, the authors showed that BEC subpopulations preserve metagene expression and the ability to form neighborhoods in the absence of mesenchymal cells, suggesting that an epithelial-autonomous mechanism might be sufficient to generate and/or maintain BEC heterogeneity.

In line with the description of a Technique and Resource Article, the manuscript provides a wealth of information about the transcriptional profile and spatial distribution of BEC subpopulations in control and BDL mouse livers and offers makers that might help the field follow the fate of these subpopulations in various disease contexts. The combination of transcriptomics, multi-channel in situ hybridization, sophisticated image analysis, and in vitro culture of primary biliary cells is impressive. As described below, I have some concerns about the strategies used for clustering and choosing the markers.

Major points

Lines 113-17, "sham and BDL samples clustered separately from one another, implying that cholestasis has a greater effect than subpopulation identity on BEC transcriptomic heterogeneity. Individual samples were uniformly distributed throughout sham and BDL clusters, suggesting that clustering was not driven by variation between biological replicates (Supplemental Figure 2F)." The observed separation is rather stark. Although real biological difference might underlie this observation, I am not convinced that based on clustering of a total of four samples, a batch/sample effect can be ruled out as the basis for the observed separation of sham and BDL clusters. The authors should provide additional evidence that the separation reflects biology rather than technical variation. One straightforward test would be Harmony integration based on sample_id, which can show whether the corresponding sham and BDL clusters merge upon batch correction or remain separate. If they indeed merge, even with a low theta (gentle correction), this would strongly suggest that batch effect led to the full separation. This would not mean that BDL does not affect BEC transcriptome, but it will sharpen the focus on those DEGs that are truly due to cholestasis.

Marker identification with NS-Forest. While the logic of why the authors chose to use NS-Forest is described clearly, I am not confident that this package has actually identified the best possible markers to distinguish the clusters. If in Supplemental Table 2, one sorts the genes based on Δpct (pct1 - pct2) and then based on cluster name, Fam107b ranks #1 for Sham 1 with the highest Δpct and a high log2FC. Palld, however, is ranked #11 for Sham 1, with a lower Δpct and a much lower log2FC. And the violin plot shown in Supplemental Figure 4A suggests a lower power for this gene to distinguish Sham1 from others, especially Sham 3. Ets1, Ngf, and Myc all look like better options to distinguish Sham 1 from all other clusters. Lack of statistical analysis makes it difficult to assess the ability of Palld to distinguish Sham 1 from others. I am most surprised by the selection of a single marker, Fos, for Sham 2. Fos is ranked as #2 in the Sham 2 group, with Txnip ranking #1. However, the Fox2 Δpct is only 0.264 (lower than Fam107b). For Sham 3 and Sham 4, the genes selected by NS-Forest are among the top three of each group based on

Δpct. A huge amount of work has gone into the 7-channel HCR-FISH and the following quantifications, so I am not suggesting that the authors should test additional markers. However, I don't see strong evidence here that NS-Forest is performing better than a simple alignment of the markers based on Δpct and in fact it might not be as good. Generating composite feature plots and violin plots like Figure 2C-F for Ets1+Fam107b (Sham 1) and Fos+Txnip (Sham 2) might indicate that those combinations would have been better choices (or confirm that NS-Forest is superior to selection based on Δpct rank).

Figure 3B: The classifications shown in this figure do not match the expected presence of relatively high levels of the corresponding metagene. For example, the cell marked as metagene 4 does not appear to have more red dots compared to other cells in this bile duct and instead has a relatively large number of dots for the three other metagenes. Can the authors show images that are better representatives for their quantifications?

Lines 269-273: Using one or two genes per cluster to combine BDL clusters does not use the wealth of information available for gene expression in these clusters. I do not find the reason that the authors have provided for this decision particularly convincing ("to maintain a conservative clustering strategy and avoid overrepresenting control-defined BEC populations"): After all, this is based on a decision made by the authors earlier in the manuscript to use the Sham samples to identify the Metagenes. If the transcriptional profiles of Sham and BDL cells are so different that they fully segregate into different clusters, perhaps a different set of Metagenes would best distinguish the BDL clusters from one another.

Additional points

Figure 1A: Is the description of white and yellow arrowheads in the legend switched?

Figure 1B: The choice of colors is not ideal. Some colors are close to each other, especially BDL2 and BDL7. It is not easy to come up with 6-7 easily distinguishable colors along a single color scale, so perhaps a different color scale can be used for the hepatocytes.

Please use the official gene symbol of Epcam instead of Cd326, as it's somewhat confusing.

Supplemental Figure 2J: It is not clear how the authors reached the conclusion that BDL1 and BDL3 showed the highest enrichment for TGF-ß. Level of TGF-ß in BDL2 and BDL7 seems to be comparable to that in BDL3. Without statistical analysis, it becomes difficult to reach such conclusions unless the difference is very clear (like ECM in BDL7).

Line 111: Supplemental Figure 1C-D instead of 2C-D?

Line 112: Supplemental Figure 1E instead of 2E? And the same for line 117 (1F)

Line 244: Consider "to gain insight into the …"

Line 247: "are linked to BEC identity"

Line 280: This should probably be Supplemental Tables 4-7.

Lines 311-312, "Metagenes 1-3 exhibited enrichment in a single subcluster, while Metagene 4 was reliably expressed in BDL-5 (Figure 5D)." Based on the plots and the figure legend, I am not sure whether this sentence describes the data in Figure 5D clearly / correctly. Should it be "was not reliably expressed in BDL-5"?

Lines 316-328: Some of the reference to Figure 6A-D seem to be incorrect.

Lines 359-361: It is not clear whether the authors consider the broad expression of Hnf4a and especially Alb across all BEC clusters as biologically meaningful or an artefact, potentially related to the presence of periportal hepatocytes in their samples. Please explicitly mention.

<u>Reviewer 2</u>

The manuscript by Kanakanui seeks to explore the heterogeneity within the biliary tree. They use a SOX9-EGFP reporting mouse to enrich for rare biliary epithelial cells within the bile duct and then compare the heterogeneity of the cells between wild type and bile duct ligated animals. The authors define and validate a number of metagene signatures to characterise biliary heterogeneity in the mouse and explore how this heterogeneity is spatially patterned.

On the whole the manuscript is well written and clear. I also applauded the authors for taking on this challenge. There has been a paucity of high-quality markers to define heterogeneity within the biliary tree and as the authors demonstrate here, many of the canonical markers that are used to define large versus small ducts are ubiquitously expressed in single cell datasets. Whether or not this is concordant with protein is unclear, yet when considering how we define single cell and nuclear data having these types of atlases are important for standardisation and interpretation of future data.

Major comments

1. As the authors identify, classical clustering methods struggle to identify heterogeneity within highly homogenous transcriptional cell states (such as within the biliary epithelium). Did the authors consider applying any alternate algorithms that do not rely on classical clustering to their data? I'm particularly thing of probabilistic approaches such as recently published STATOR or latent space models such as SCVI, SCANVI or TotalVI? Could these identify heterogeneity with more granularity and avoid the concerns of over/under-clustering? If not, then perhaps discuss in the text how some of these emerging analyses could give improved resolution albeit they are not benchmarked against current methods.

2. When does heterogeneity emerge within the biliary tree? I appreciate that this paper explores adult livers and regeneration following by BDL, however have the authors looked at late embryonic or early postnatal tissue to define whether they see their different metagene signatures here? I ask because there are recent data suggesting that zonation within the hepatocytes emerges very late and development and then becomes more fixed through early neonatal life - it would be interesting to understand whether this is true of other hepatic epithelial cells?

3. While the observation that the proportions of Metagene states changes in bile duct ligated livers what is the evidence this is applicable across cholestatic models? Have the authors explored tissue from MDR2-knockouts, DDC, TAA or ANIT treatments to ask whether the same changes in cell populations and representation occurs here? Otherwise is there a risk that this is simply phenomenology and cell state changes that are specific to BDL, but not generalisable to cholestasis?

Minor comments

1. Could the authors include a more robust rationale within the text or indeed within the figure as to why they have excluded Pecam1-positive cells given this is a paper about cellular heterogeneity? I think it's important that there is a very clear rationale for the removal of this group (I don't disagree with the approach, just think it should be very clearly defined as to why).

Reviewer 3

SUMMARY OF THE ADVANCE MADE IN THIS PAPER AND ITS POTENTIAL SIGNIFICANCE TO THE FIELD

The information provided in this resource article is crucial to the general field of cell lineage heterogeneity and specifically to liver injury repair/response involving intrahepatic biliary epithelial cell (BEC) biology. The deeply characterized BEC single cell atlas provided in this manuscript will be unique for truly normal liver with the added and compared experimental bile duct ligation (BDL) injury response BEC single cell atlas. The authors not only took advantage of their Sox9EGFP+ mouse line to isolate BECs and thereby critically increase the number of BECs interrogated, but also used existing omic platforms in unique ways, and importantly compared and discussed published human and mouse data sets defining four BEC subtypes. Finally, the authors use HCR FISH to spatially localize their identified metagene-defined BEC subtypes in vivo and in vitro. Therefore, the two approaches and information provided in this resource article greatly strengthens the quality and rigor of the manuscript.

Interestingly, the authors found that BECs belonging to a given subpopulation were most likely to have neighbors of the same identity in vitro and in vivo. These results indicate that subpopulations of BECs form homogenous cellular clusters that were found to be located throughout the IHBD (intrahepatic bile duct) system suggesting that IHBDs are overall heterogeneous. Additionally, 1. All the defined subpopulations of BECs in this study were found in vitro, and 2. Given the spatial cluster distribution of the BEC subtypes in vitro invokes an epithelial-autonomous regulation warranting future studies.

Using the BDL injury model the authors found 1. downregulated subtype specific gene expression and upregulated damage associated transcriptional profiles across all subtypes, and 2. a proliferative subpopulation that was found in three of the four metagenes. This suggests an interesting transcriptional injury response wherein cholestasis impacts and overrides part of the BEC subtype and cell identity signature.

SUGGESTIONS TO AUTHORS

Minor concerns:

1. Essential - Does the differential Sox9 expression level associate with BEC subpopulation clusters/localization of heterogeneous subpopulations? More specifically, 1. Does the linked Sox9EGFP level and the scRNA-seq Sox9 level correlate, and 2. Does the Sox9 (either GFP surrogate and/or mRNA) level associate with a specific BEC subpopulation? Some information is provided in the current manuscript for association of GFPlow and BEC-III.

2. Essential - This resource article begins to touch on a controversy in the field related to differences in molecular characterization of duct lumenal size versus size of the BEC itself. "We also examined expression of reported small ductule and large duct markers, which represent classical markers of BEC heterogeneity [4, 6, 16-18]. While the expression of small BEC marker Hrh1 was enriched in Sham 3 and BDL 3-4, other markers were either broadly expressed in BEC clusters or exhibited rare, scattered detection across clusters (Supplemental Figure 3B & C). " Absolutely critical to be clear when inferring duct lumenal size versus size of the BEC.

3. Essential/Extension - With the data in hand (HCR FISH), the authors have the ability to determine if there is an association with a signature (BEC subtype) and actual BEC size? Take advantage of Epcam segmentation to assess size of each BEC and determine if a metagene/subtype expression in vivo or in vitro is associated with a specific range of BEC size - "While correlation between BEC subpopulation and IHBD size did not reach significance, BEC-III exhibited a weak correlation with larger ducts and BEC-IV with smaller ductules (Figure 3D)."

4. Extension - Potential to further clear up the size debate - given the controversy of markers for large and small sized lumenal ducts and the interesting clustering of BEC subpopulations, to verify the data in Supplemental Figure 2 - "suggesting that BEC identity associated with anatomical location in the IHBDs is maintained in both homeostasis and injury (Supplemental Figure 2K)." - opportunity to perform HCR FISH.

5. Essential - Interesting molecular factor pathway differential expression in subpopulations. What are the lists of genes associated with Supplemental Figure 2 - ligands, receptors, effectors? Does this fit with the in vitro finding that "BEC heterogeneity is regulated in an epithelial-autonomous manner"?

First revision

<u>Author response to reviewers' comments</u>

Reviewer #1 (Remarks to the Author):

In manuscript dev.205690, Kanakanui and colleagues set out to identify biliary epithelial cell (BEC) subpopulations during homeostasis and after liver injury and biomarkers that can distinguish those subpopulations. Previous work by the authors and others has established heterogeneity in BECs (for example, based on the expression level of Sox9). However, in scRNA- seq datasets, BECs are almost

always grouped in a single cluster. This suggests that the transcriptional difference among BEC subpopulations is not sufficient to lead to the identification of distinct BEC clusters when analyzed in the context of the whole liver. Given the involvement of BECs in the pathophysiology of a host of liver diseases, it is important to be able to unambiguously identify BECs subpopulations and their distribution in control and disease livers, highlighting the value of identifying makers that can reliably distinguish them.

To address this unmet need, the authors took advantage of a Sox9-EGFP alleles from their previous work to isolate Sox9+ cells from adult mouse livers, with or without bile duct ligation (BDL) as a form of cholestatic liver injury and subjected the isolated cells to scRNA-seq. The authors found that BECs from control and BDL livers fall into distinct clusters. Analysis of known hepatobiliary markers and DEGs along with GSEA showed that the control and BDL clusters are indeed transcriptionally distinct but failed to identify a manageable number of specific biomarkers that exhibit a binary expression for each cluster. Therefore, the authors applied a machine learning algorithm called NS-Forest and identified minimal biomarker sets composed of 1-2 genes for each cluster (termed metagenes). Using a 7-panel HCR-FISH covering all the markers in the above- mentioned metagenes, the authors analyzed the spatial distribution of the four BEC subpopulations in control livers and found that 1) Each duct contains at least two different BEC subpopulations, 2) Some BECs preferentially populate ducts of a certain size, and 3) BEC subpopulations tend to form neighborhoods. Using the metagenes, the authors reassigned BEC clustering in the BDL liver and identified those BDL clusters that are capable of entering the cell cycle upon cholestatic injury. Finally, using in vitro cultures of primary BECs established from wild-type bile duct fragments, the authors showed that BEC subpopulations preserve metagene expression and the ability to form neighborhoods in the absence of mesenchymal cells, suggesting that an epithelial-autonomous mechanism might be sufficient to generate and/or maintain BEC heterogeneity.

In line with the description of a Technique and Resource Article, the manuscript provides a wealth of information about the transcriptional profile and spatial distribution of BEC subpopulations in control and BDL mouse livers and offers makers that might help the field follow the fate of these subpopulations in various disease contexts. The combination of transcriptomics, multi-channel in situ hybridization, sophisticated image analysis, and in vitro culture of primary biliary cells is impressive. As described below, I have some concerns about the strategies used for clustering and choosing the markers.

#1. Lines 113-17, "sham and BDL samples clustered separately from one another, implying that cholestasis has a greater effect than subpopulation identity on BEC transcriptomic heterogeneity. Individual samples were uniformly distributed throughout sham and BDL clusters, suggesting that clustering was not driven by variation between biological replicates (Supplemental Figure 2F)." The observed separation is rather stark. Although real biological difference might underlie this observation, I am not convinced that based on clustering of a total of four samples, a batch/sample effect can be ruled out as the basis for the observed separation of sham and BDL clusters. The authors should provide additional evidence that the separation reflects biology rather than technical variation. One straightforward test would be Harmony integration based on sample_id, which can show whether the corresponding sham and BDL clusters merge upon batch correction or remain separate. If they indeed merge, even with a low theta (gentle correction), this would strongly suggest that batch effect led to the full separation. This would not mean that BDL does not affect BEC transcriptome, but it will sharpen the focus on those DEGs that are truly due to cholestasis.

RESPONSE: *We appreciate the reviewer's suggestion of attempting a gentle correction. To minimize potential batch effects in our data, samples were dissociated, FACS-isolated, and fixed as two sets of sham and BDL "pairs". Split-pool library preparation was carried out on all four samples at the same time using the same kit from Parse Biosciences. Finally, all samples shown were sequenced together. Because technical sources of batch variation were minimized by our wet lab approach, we reasoned that best practices would entail not performing computational integration. We did make a cursory attempt at integration when first analyzing our data, but did not carefully pursue it. By request of the reviewer, we performed Harmony Integration with a range of theta values (θ = 0, 1, 2, 3, 4) and found even with stringent correction (up to θ = 4), sham and BDL conditions had minimal overlap and remained distinct groups. To simplify data presentation in the manuscript, we added a representative UMAP plot depicting the separation in the two conditions (sham and BDL) at a relatively stringent correction (θ = 3) (Fig. S1G) and include the results of other theta values below.*

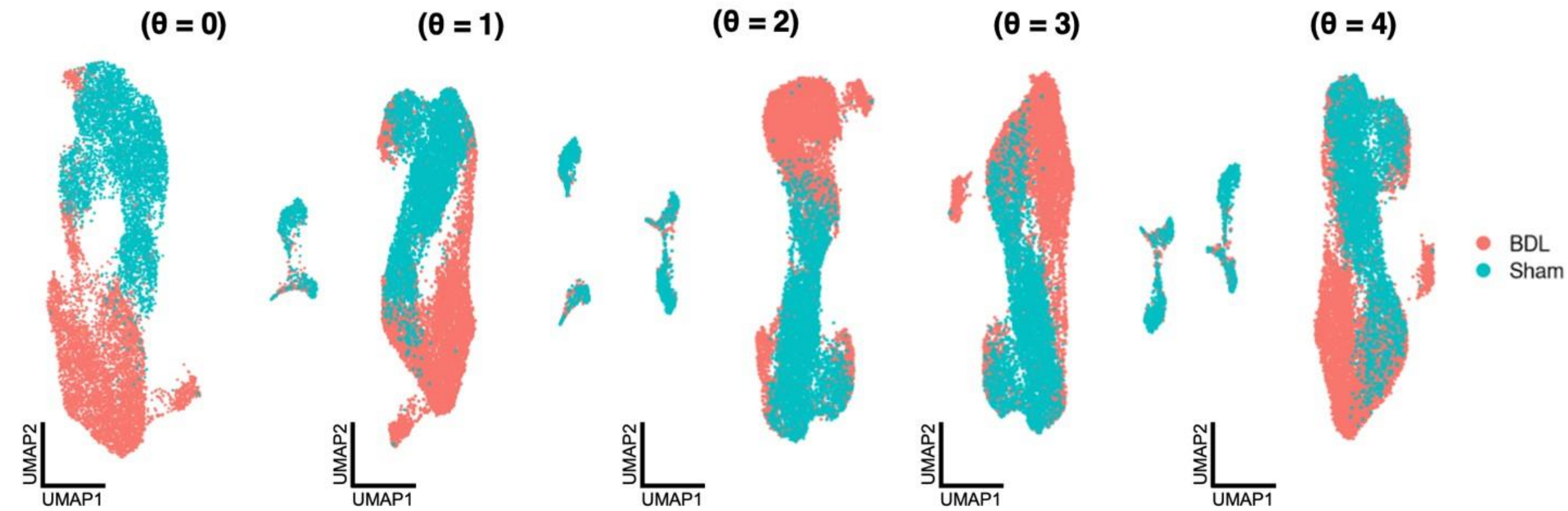

#2. Marker identification with NS-Forest. While the logic of why the authors chose to use NS- Forest is described clearly, I am not confident that this package has actually identified the best possible markers to distinguish the clusters. If in Supplemental Table 2, one sorts the genes based on Δpct (pct1 - pct2) and then based on cluster name, Fam107b ranks #1 for Sham 1 with the highest Δpct and a high log2FC. Palld, however, is ranked #11 for Sham 1, with a lower Δpct and a much lower log2FC. And the violin plot shown in Supplemental Figure 4A suggests a lower power for this gene to distinguish Sham1 from others, especially Sham 3. Ets1, Ngf, and Myc all look like better options to distinguish Sham 1 from all other clusters. Lack of statistical analysis makes it difficult to assess the ability of Palld to distinguish Sham 1 from others. I am most surprised by the selection of a single marker, Fos, for Sham 2. Fos is ranked as #2 in the Sham 2 group, with Txnip ranking #1. However, the Fox2 Δpct is only 0.264 (lower than Fam107b). For Sham 3 and Sham 4, the genes selected by NS-Forest are among the top three of each group based on Δpct. A huge amount of work has gone into the 7-channel HCR-FISH and the following quantifications, so I am not suggesting that the authors should test additional markers. However, I don't see strong evidence here that NS-Forest is performing better than a simple alignment of the markers based on Δpct and in fact it might not be as good. Generating composite feature plots and violin plots like Figure 2C-F for Ets1+Fam107b (Sham 1) and Fos+Txnip (Sham 2) might indicate that those combinations would have been better choices (or confirm that NS-Forest is superior to selection based on Δpct rank).

RESPONSE: *This is an excellent suggestion and a good way to test "manually" identified alternative metagenes. In the revised manuscript, we apply the Δpct analysis suggested by the reviewer, and list the top 10 alternative genes for each sham BEC cluster in Table S3. We also include the feature/violin plots as well as the single gene plots for the specific genes requested by the reviewer (Fig. S4H-K). We have revised the manuscript as follows:*

"To further evaluate our marker selection strategy, we applied an alternative approach for detecting genes with binary expression. We computed the difference in the proportion of cells expressing a given gene in one sham BEC cluster compared to the other three sham BEC clusters *(Δpct=pct1-* pct2). Using this approach, we identified the top 10 genes for each sham BEC cluster based on ranked Δpct (Table S4). As Metagenes 1 and 2 exhibited the least binary expression patterns, we specifically examined alternatives for the broadly expressed gene in each (*Palld* and *Fos*, respectively) and generated new composite plots based on the highest Δpct value (Fig. S4H-K). Relative to NSForest-selected candidates, metagenes containing alternative genes (*Ets1* and *Myc* for Metagene 1 and *Txnip* for Metagene 2) demonstrated greater enrichment within the "target" cluster, but reduced specificity (Fig. S4H-I,K). For this reason, we proceeded to apply NSForest- selected metagenes throughout our study."

#3. Figure 3B: The classifications shown in this figure do not match the expected presence of relatively high levels of the corresponding metagene. For example, the cell marked as metagene 4 does not appear to have more red dots compared to other cells in this bile duct and instead has a relatively large number of dots for the three other metagenes. Can the authors show images that are better representatives for their quantifications?

RESPONSE: *The method for z-scoring metagene expression by HCR FISH (as well as cell-cell variability in whether FISH signal appears as large/focal puncta vs. distributed signal) does make it challenging to*

visually assign metagene identity. We have included better representative images for Fig. 3B in the revised manuscript which we hope more clearly represent metagene expression.

#4. Lines 269-273: Using one or two genes per cluster to combine BDL clusters does not use the wealth of information available for gene expression in these clusters. I do not find the reason that the authors have provided for this decision particularly convincing ("to maintain a conservative clustering strategy and avoid overrepresenting control-defined BEC populations"): After all, this is based on a decision made by the authors earlier in the manuscript to use the Sham samples to identify the Metagenes. If the transcriptional profiles of Sham and BDL cells are so different that they fully segregate into different clusters, perhaps a different set of Metagenes would best distinguish the BDL clusters from one another.

RESPONSE*: We understand the reviewer's concern. While we agree that a different set of metagenes could potentially better distinguish BDL clusters, the primary goal of this paper is to identify biomarkers for BEC subpopulations that can then be applied universally to further define BEC heterogeneity in different contexts. We demonstrate proof of concept for this approach by identifying subpopulations enriched for metagenes found in sham clusters in our BDL samples, rather than defining new BDL-specific metagenes. Reviewer 2 raised a concern, below, that subpopulations observed in different contexts could represent "phenomenology" as opposed to biological "ground truth". We would argue that "matching" BDL cluster identities to metagenes identified in Sham samples helps mitigate the risk of pursuing phenomenology and increases the probability that our manuscript presents a framework that will be applicable to other biological contexts (injury models, developmental timepoints, etc).*

The reviewer is correct that merging BDL 3 and BDL 4 discards some degree of heterogeneity that is likely biologically interesting. To acknowledge and include this heterogeneity in the revised manuscript, we added new differential expression and a gene set enrichment analyses comparing BDL 3 and BDL 4 (Fig. S6B-C). We also refined our rationale for merging BDL 3 and BDL 4 in the revised manuscript, as follows:

"Interestingly, Metagene 3 was enriched in both BDL 3 and BDL 4 (Figs 4A, S6A). To characterize the transcriptomic differences between these subpopulations, we performed differential gene expression analysis and identified 447 DEGs (Table S6). GSEA revealed that BDL 3 is enriched for inflammation-associated gene sets, while BDL 4 is enriched for terms related to translation and ribosome assembly (Fig. S6C). Despite significant gene expression differences, the shared enrichment of Metagene 3 in both clusters implies that they may have derived from BECs represented by Sham 3 (Fig. S6A-C). Therefore, in line with our goal to compare uninjured control BEC subpopulations with their cognate injured populations, we merged BDL 3 and BDL 4 into a single cluster for downstream analyses."

Because Reviewer 1 also suggested attempting integration of Sham and BDL clusters using Harmony, we decided to leverage this approach as an alternative method for matching BDL clusters to analogous sham clusters. We selected a stringent integration setting (θ = 3) and compared the original sham and BDL clusters to new integrated clusters, visualizing the data by Sankey plot (below). Most cells in BDL 3 along with a small number of cells from BDL 4 are assigned to an integrated cluster which also includes most of the cells from Sham 3 ("Harmony 0"). However, the remainder of the cells from BDL 4 are split between an integrated cluster containing part of Sham 2 (Harmony 4) and an integrated cluster containing only BDL-derived cells (Harmony 2). We note that this analysis relies on relatively stringent integration parameters and believe it highlights the complexity of matching BEC subpopulation identities between control and injury conditions. Definitive identification of what injury-specific cell states derive from which homeostatic BEC subpopulations will require advanced lineage tracing techniques outside of the scope of the present study. This is now addressed more extensively in the Discussion:

"Because computational identification of cellular subpopulations in scRNA-seq data analysis is susceptible to over-clustering, we prioritized a conservative approach that would also facilitate comparison of Sham and BDL subpopulations. We also reasoned that, given the lack of substantial hepatocyte-to-BEC transdifferentiation following BDL, all BDL BEC subpopulations were most likely to derive from BEC subpopulations present in sham controls. Therefore, shared enrichment of Metagene 3 could suggest that the BDL clusters we chose to merge were both derived from BEC-III. However, definitively proving precise relationships between BEC subpopulations in homeostasis and following injury will require advanced lineage tracing techniques and is beyond the scope of the current study. Another important consideration is whether other models of cholestatic or hepatocellular liver injury

will induce biological responses conducive to application of the metagenes described here, or if biliary heterogeneity varies substantially in different contexts. Future studies may be able to further resolve BEC transcriptomic heterogeneity in different injury and disease models, using the metagene-defined subpopulations identified here as a foundation.”

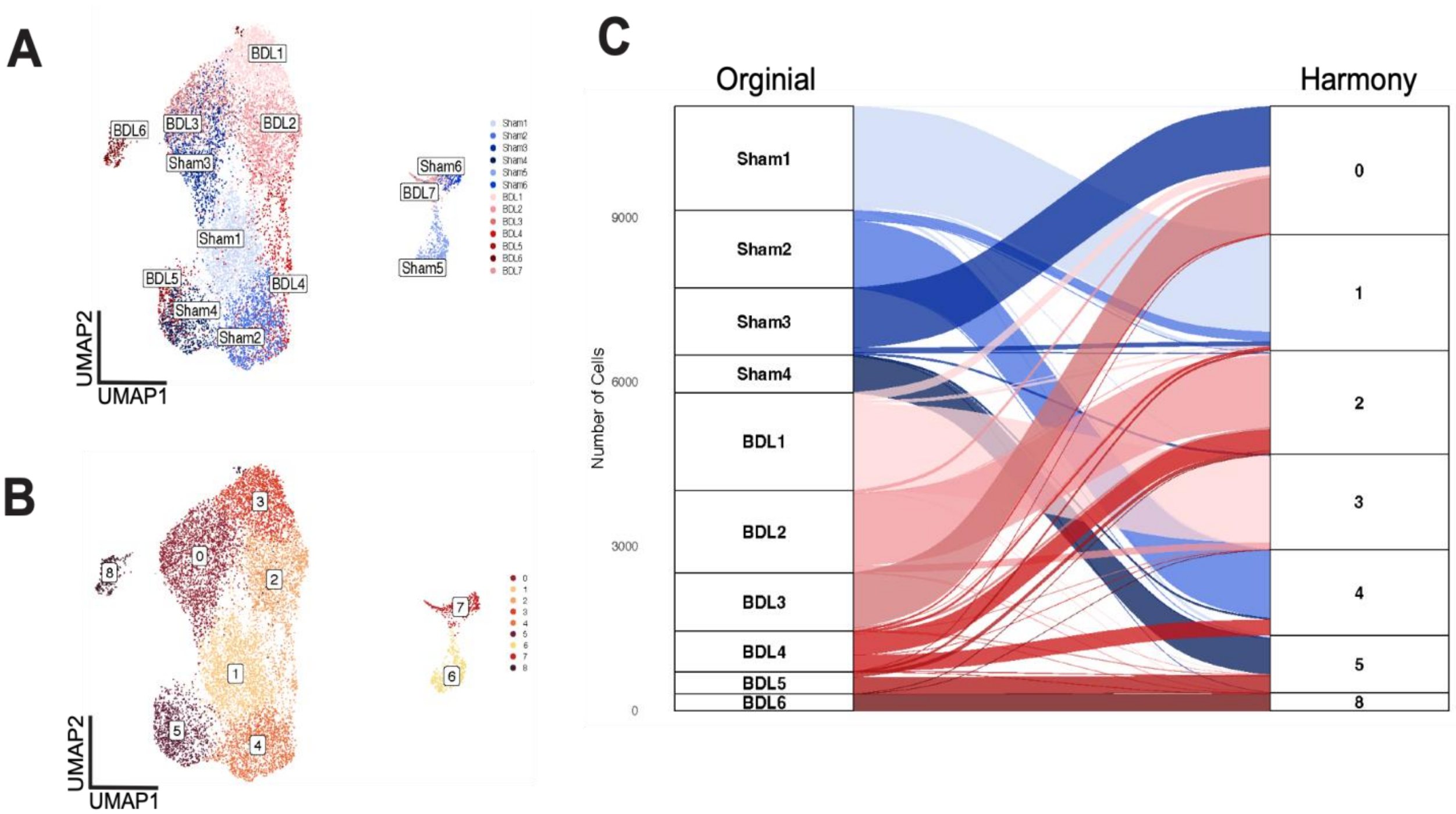

Additional points

Figure 1A: Is the description of white and yellow arrowheads in the legend switched?

RESPONSE: *Thank you for catching this error. We have changed this in the revised manuscript to correctly label BECs (yellow) and hybrid hepatocytes (white).*

Figure 1B: The choice of colors is not ideal. Some colors are close to each other, especially BDL2 and BDL7. It is not easy to come up with 6-7 easily distinguishable colors along a single color scale, so perhaps a different color scale can be used for the hepatocytes.

RESPONSE: *As there was only one UMAP including the HybHeps, which are close in color to the BEC clusters, we kept the color scheme the same throughout the paper and revised the UMAP with the HybHeps (Fig. 1B) to include the cluster labels next to the corresponding cluster.*

Please use the official gene symbol of Epcam instead of Cd326, as it's somewhat confusing.

RESPONSE: *We revised the manuscript to replace instances of “Cd326/CD326” with Epcam/*EPCAM.

Supplemental Figure 2J: It is not clear how the authors reached the conclusion that BDL1 and BDL3 showed the highest enrichment for TGF-ß. Level of TGF-ß in BDL2 and BDL7 seems tobe comparable to that in BDL3. Without statistical analysis, it becomes difficult to reach such conclusions unless the difference is very clear (like ECM in BDL7).

RESPONSE: *We appreciate and agree with the reviewer’s rationale for this comment and have removed the claim from the revised manuscript.*

Line 111: Supplemental Figure 1C-D instead of 2C-D?
Line 112: Supplemental Figure 1E instead of 2E? And the same for line 117 (1F) Line 244:
Consider "to gain insight into the …"

Line 247: "are linked to BEC identity"
Line 280: This should probably be Supplemental Tables 4-7.
Lines 311-312, "Metagenes 1-3 exhibited enrichment in a single subcluster, while Metagene 4 was reliably expressed in BDL-5 (Figure 5D)." Based on the plots and the figure legend, I am not sure whether this sentence describes the data in Figure 5D clearly / correctly. Should it be "was not reliably expressed in BDL-5"?
Lines 316-328: Some of the reference to Figure 6A-D seem to be incorrect.

***RESPONSE**: We thank the reviewer for providing a detailed correction list (Line 111…Line 316-328) and have corrected these errors in the revised manuscript.*

Lines 359-361: It is not clear whether the authors consider the broad expression of Hnf4a and especially Alb across all BEC clusters as biologically meaningful or an artefact, potentially related to the presence of periportal hepatocytes in their samples. Please explicitly mention.

***RESPONSE**: This is an important point and we appreciate the reviewer highlighting it; we consider this to be biologically meaningful and not a technical artifact. We acknowledge it is possible that RNA from periportal hepatocytes could "spill" into BEC samples during single cell library prep, but consider it unlikely that this would have happened at the scale required to result in the observed Hnf4a and Alb expression patterns. Additionally, previously published data from several groups demonstrate "canonical" hepatocyte gene expression in BECs, including Hnf4a, Alb, and Ttr . We also observed this in our prior bulk RNA-seq assays on BECs, finding that Ttr was specifically enriched in Sox9$^{EGFP\text{-}high}$ BECs (Tulasi et al., 2019). We have addressed this in the revised manuscript:*

"While it is possible that the presence of HybHeps in our preps resulted in the contamination of BECs with hepatocyte transcripts, this seems unlikely given: (1) the broad detection of Hnf4a and Alb and (2) the presence of these and other hepatocyte-associated transcripts in other published scRNA-seq of mouse BECs (Planas-Paz et al., 2019; Pu et al., 2023)."

Reviewer #2 (Remarks to the Author):
The manuscript by Kanakanui seeks to explore the heterogeneity within the biliary tree. They use a SOX9-EGFP reporting mouse to enrich for rare biliary epithelial cells within the bile duct and then compare the heterogeneity of the cells between wild type and bile duct ligated animals. The authors define and validate a number of metagene signatures to characterise biliary heterogeneity in the mouse and explore how this heterogeneity is spatially patterned.
On the whole the manuscript is well written and clear. I also applauded the authors for taking on this challenge. There has been a paucity of high-quality markers to define heterogeneity within the biliary tree and as the authors demonstrate here, many of the canonical markers that are used to define large versus small ducts are ubiquitously expressed in single cell datasets. Whether or not this is concordant with protein is unclear, yet when considering how we define single cell and nuclear data having these types of atlases are important for standardisation and interpretation of future data.

#1. As the authors identify, classical clustering methods struggle to identify heterogeneity within highly homogenous transcriptional cell states (such as within the biliary epithelium). Did the authors consider applying any alternate algorithms that do not rely on classical clustering to their data? I'm particularly thing of probabilistic approaches such as recently published STATOR or latent space models such as SCVI, SCANVI or TotalVI? Could these identify heterogeneity with more granularity and avoid the concerns of over/under-clustering? If not, then perhaps discuss in the text how some of these emerging analyses could give improved resolution albeit they are not benchmarked against current methods.

***RESPONSE**: This is an excellent suggestion, and we thank the reviewer for calling our attention to these alternatives to conventional clustering. Because a recent preprint used STATOR on biliary epithelium, we selected this package to apply as a test on our data (Jarman et al., 2025). Weattempted running STATOR (v1.1) using several different settings and chose to analyze our scRNA- seq dataset using the top 1,000 highly variable genes (Jansma et al., 2025). This maximized the number of genes considered while working within the limits of our computational resources. STATOR identified 6 cell "states", with most cells falling into a single state ("Stator 2"). Comparison of our original clustering with the STATOR groups revealed no clear correspondence (below). More importantly, cells from each cluster dispersed across many STATOR-defined states. Because these results are difficult to interpret*

and likely require extensive technical optimization and independent validation to understand, we have omitted them from the revised manuscript but include them here.

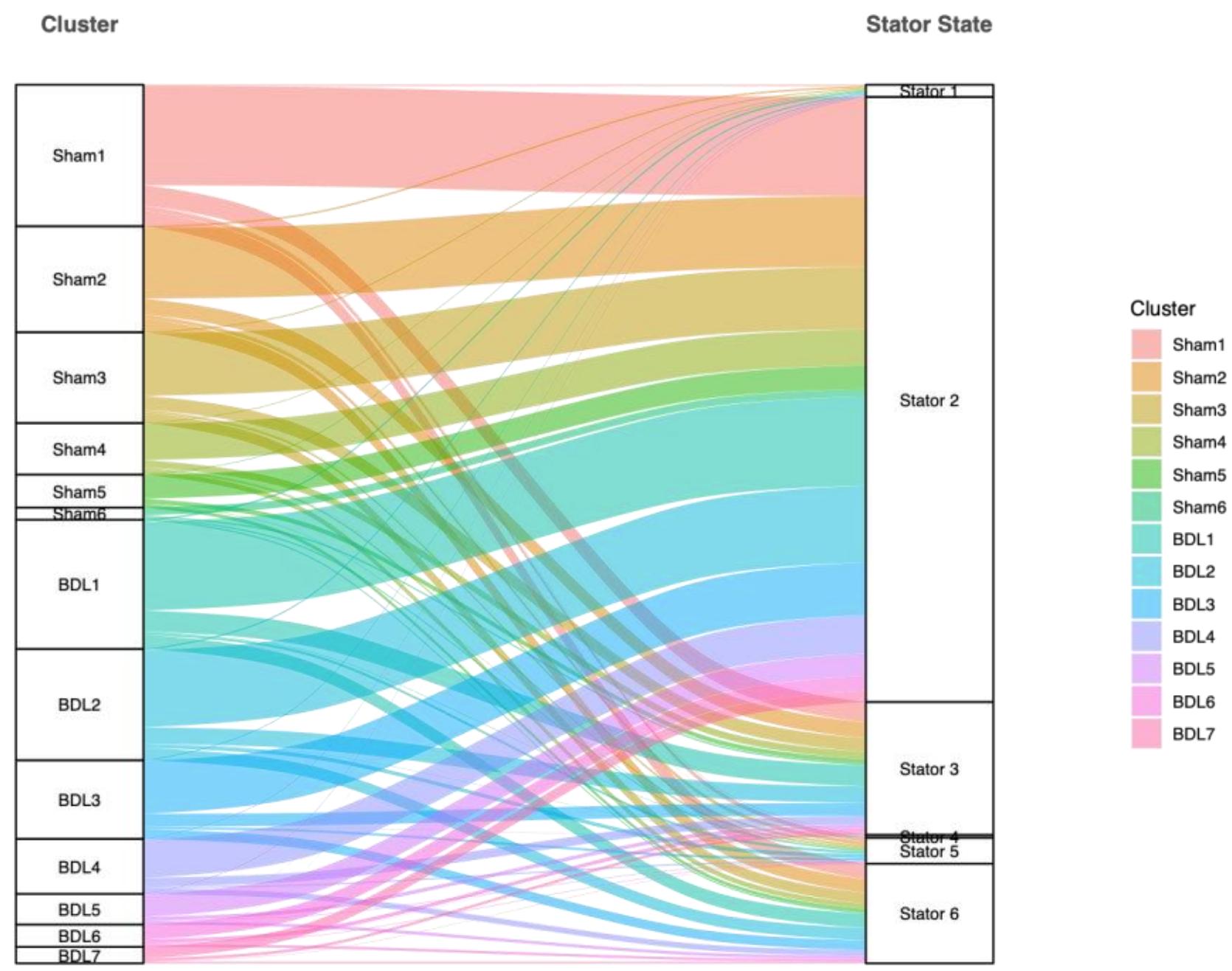

#2. When does heterogeneity emerge within the biliary tree? I appreciate that this paper explores adult livers and regeneration following by BDL, however have the authors looked at late embryonic or early postnatal tissue to define whether they see their different metagene signatures here? I ask because there are recent data suggesting that zonation within the hepatocytes emerges very late and development and then becomes more fixed through early neonatal life - it would be interesting to understand whether this is true of other hepatic epithelial cells?

***RESPONSE**: While we share the reviewer's excitement for this subject, our primary focus was to define biomarkers in post-development homeostasis that can be applied to look at different disease models in future work. We did not feel like our study provides significant direct insight on biliary development that would allow us to meaningfully speculate on the subject in the revised manuscript. We do agree that determining when BEC heterogeneity arises is an important question for the field and are hopeful that our identification of Metagene signatures for adult BEC populations can be applied to generate future insight for liver development.*

#3. While the observation that the proportions of Metagene states changes in bile duct ligated livers what is the evidence this is applicable across cholestatic models? Have the authors explored tissue from MDR2-knockouts, DDC, TAA or ANIT treatments to ask whether the same changes in cell populations and representation occurs here? Otherwise is there a risk that this is simply phenomenology and cell state changes that are specific to BDL, but not generalisable to cholestasis?

***RESPONSE**: The reviewer brings up an excellent point, and we have revised the Discussion as shown below to acknowledge this limitation of our study. We have not yet conducted scRNA-seq in other damage models and agree these studies will be necessary to assess how widely applicable our metagenes are. However, as highlighted in our response to Reviewer 1's concerns about merging BDL clusters enriched for Metagene 3, our general approach was to anchor our definition of BDL clusters relative to heterogeneity observed in uninjured controls. We believe this reduces the risk of phenomenology relative to identifying new metagenes for BDL clusters. It is possible that different injury modalities might elicit distinct biological responses, but as long as these involve some continued expression of metagenes present in healthy BECs, it should be possible to identify subpopulations using the approach described in our manuscript.*

"Another important consideration is whether other models of cholestatic or hepatocellular liver injury will induce biological responses conducive to application of the metagenes described here, or if biliary heterogeneity varies substantially in different contexts."

#4. Could the authors include a more robust rationale within the text or indeed within the figure as to why they have excluded Pecam1-positive cells given this is a paper about cellular heterogeneity? I think it's important that there is a very clear rationale for the removal of this group (I don't disagree with the approach, just think it should be very clearly defined as to why).

RESPONSE: *We appreciate the reviewer's comment and have revised the manuscript to include a clearer rationale for the exclusion of Pecam1 positive cells:*

"To focus our analysis on epithelium, we excluded a cluster of contaminating endothelial cells, marked by the canonical endothelial marker *Pecam1*, as well as clusters containing fewer than 100 cells (Fig. S1E)."

Reviewer #3 (Remarks to the Author):

#1. Essential - Does the differential Sox9 expression level associate with BEC subpopulation clusters/localization of heterogeneous subpopulations? More specifically, 1. Does the linked Sox9EGFP level and the scRNA-seq Sox9 level correlate, and 2. Does the Sox9 (either GFP surrogate and/or mRNA) level associate with a specific BEC subpopulation? Some information is provided in the current manuscript for association of GFPlow and BEC-III.

RESPONSE: *We thank the reviewer for this excellent question. We compared the expression level of Sox9 and Egfp within clusters and found no correlation between the two to delineate any potential subpopulation heterogeneity (Fig S2B). We note this along with well-documented drop- out effect in scRNA-seq in the revised manuscript. We have observed a similar lack of reliable "per cell" correlation between Sox9 and Egfp in other scRNA-seq datasets of both intestine and liver and this was our motivation to instead apply gene signatures from previously published bulk RNA- seq from sorted* GFP^{high}, GFP^{low}, *and* GFP^{sublow} *populations to assay BEC heterogeneity within our scRNA-seq dataset.*

#2. Essential - This resource article begins to touch on a controversy in the field related to differences in molecular characterization of duct lumenal size versus size of the BEC itself. "We also examined expression of reported small ductule and large duct markers, which represent classical markers of BEC heterogeneity [4, 6, 16-18]. While the expression of small BEC marker Hrh1 was enriched in Sham 3 and BDL 3-4, other markers were either broadly expressed in BEC clusters or exhibited rare, scattered detection across clusters (Supplemental Figure 3B & C). " Absolutely critical to be clear when inferring duct lumenal size versus size of the BEC.

RESPONSE: *We thank the reviewer for raising this important point regarding BEC vs. IHBD size. We have refined the language here to specify that we are referring to markers based on BEC size:*

"We also examined expression of reported <u>small BEC</u> and <u>large BEC</u> markers, which represent classical markers of BEC heterogeneity (Alpini et al., 1997b, Glaser et al., 2009, Luo et al., 2019, Francis et al., 2026, Alpini et al., 2011). While the small BEC marker *Hrh1* was enriched in Sham 3 and BDL 3-4, other markers were either broadly expressed in BEC clusters or exhibited rare, scattered detection across clusters (Fig. S3B,C)."

#3. Essential/Extension - With the data in hand (HCR FISH), the authors have the ability to determine if there is an association with a signature (BEC subtype) and actual BEC size? Take advantage of Epcam segmentation to assess size of each BEC and determine if a metagene/subtype expression in vivo or in vitro is associated with a specific range of BEC size - "While correlation between BEC subpopulation and IHBD size did not reach significance, BEC-III exhibited a weak correlation with larger ducts and BEC-IV with smaller ductules (Figure 3D)."

RESPONSE: *Following the reviewer's advice, we compared BEC size (area;*μm^2*) and IHBD size to BEC identity and found no significant relationship in vivo or in vitro. This is addressed in our revised manuscript as follows:*

"Previous studies have suggested that BEC size correlates with IHBD size, as defined by duct diameter, and that both are linked to BEC identity (Alpini et al., 1996, Ueno et al., 2003, Alpini et al., 1997a). Comparison of BEC area with both its corresponding duct diameter and subpopulation identity revealed no correlation, with small and large BECs present across ducts of varying sizes and among all subpopulations (Fig. 5SC-D). Consistent with these findings, every duct analyzed contained at least two BEC subpopulations, suggesting that no single BEC identity corresponds to a specific IHBD size (Fig. S5E)."

"As determined by our *in vivo* analyses, we found no significant correlation between cell size and BEC subpopulation identity *in vitro* (Fig. S8)."

#4. Extension - Potential to further clear up the size debate - given the controversy of markers for large and small sized lumenal ducts and the interesting clustering of BEC subpopulations, to verify the data in Supplemental Figure 2 - "suggesting that BEC identity associated with anatomical location in the IHBDs is maintained in both homeostasis and injury (Supplemental Figure 2K)." - opportunity to perform HCR FISH.

RESPONSE*: We appreciate the reviewer's suggestion to conduct HCR FISH assays in BDL samples and agree that this would be a superb extension of the manuscript. A major limitation for us at this time is that the HCR FISH reagents used now represent an older technology that has been replaced by new HCR chemistry with better signal:noise performance. The manufacturer is no longer supporting the older reagents and our stocks on hand are insufficient to complete a full complement of replicates for HCR FISH in BDL samples. This work is also currently unfunded in our lab and we lack the financial resources to purchase a full panel of "next generation" HCR FISH reagents and conduct the associated, extensive confocal microscopy.*

#5. Essential - Interesting molecular factor pathway differential expression in subpopulations. What are the lists of genes associated with Supplemental Figure 2 - ligands, receptors, effectors? Does this fit with the in vitro finding that "BEC heterogeneity is regulated in an epithelial- autonomous manner"?

RESPONSE*: We appreciate the reviewer's comment on the gene signature analyses we performed in Fig. S2, and as requested, we generated a table that lists the genes involved in each signature (Table S1). For the included pathways (TGF-ß, Yap, Notch, Hedgehog) the gene list is a mix of ligands, receptors and effectors derived from published data sets. We believe it would complicate the manuscript and only provide speculative insight to include expression data for specific sets of ligands, receptors, and effectors. However, since Notch has well-established roles in epithelial patterning and lateral inhibition, we generated gene expression plots for its relatively simple complement of ligands and receptors for purposes of responding to the reviewer (below; note that Sham 5, Sham 6, and BDL 6 all represent hybrid hepatocytes). Notably, Notch2 is the only receptor/ligand expressed significantly throughout BECs during homeostasis, while BDL induces Jag1 expression throughout BEC and Notch3 specifically in BEC-IV. It is possible that BEC organoid culture conditions induce "damage-like" gene expression – or gene expression patterns entirely unique to in vitro cultures - that play a role in maintaining BEC heterogeneity and/or spatial patterning (either including or independent of Notch signaling).*

With regards to our conclusion that BEC heterogeneity has some degree of epithelial-autonomy, BECs were grown in defined media conditions as outlined in our methods. This conclusion is based on BECs: (1) maintaining metagene heterogeneity while (2) patterning into spatially homogenous "neighborhoods" as observed in vivo, <u>without</u> a non-epithelial "niche" component and while exposed homogenously to the same media conditions.

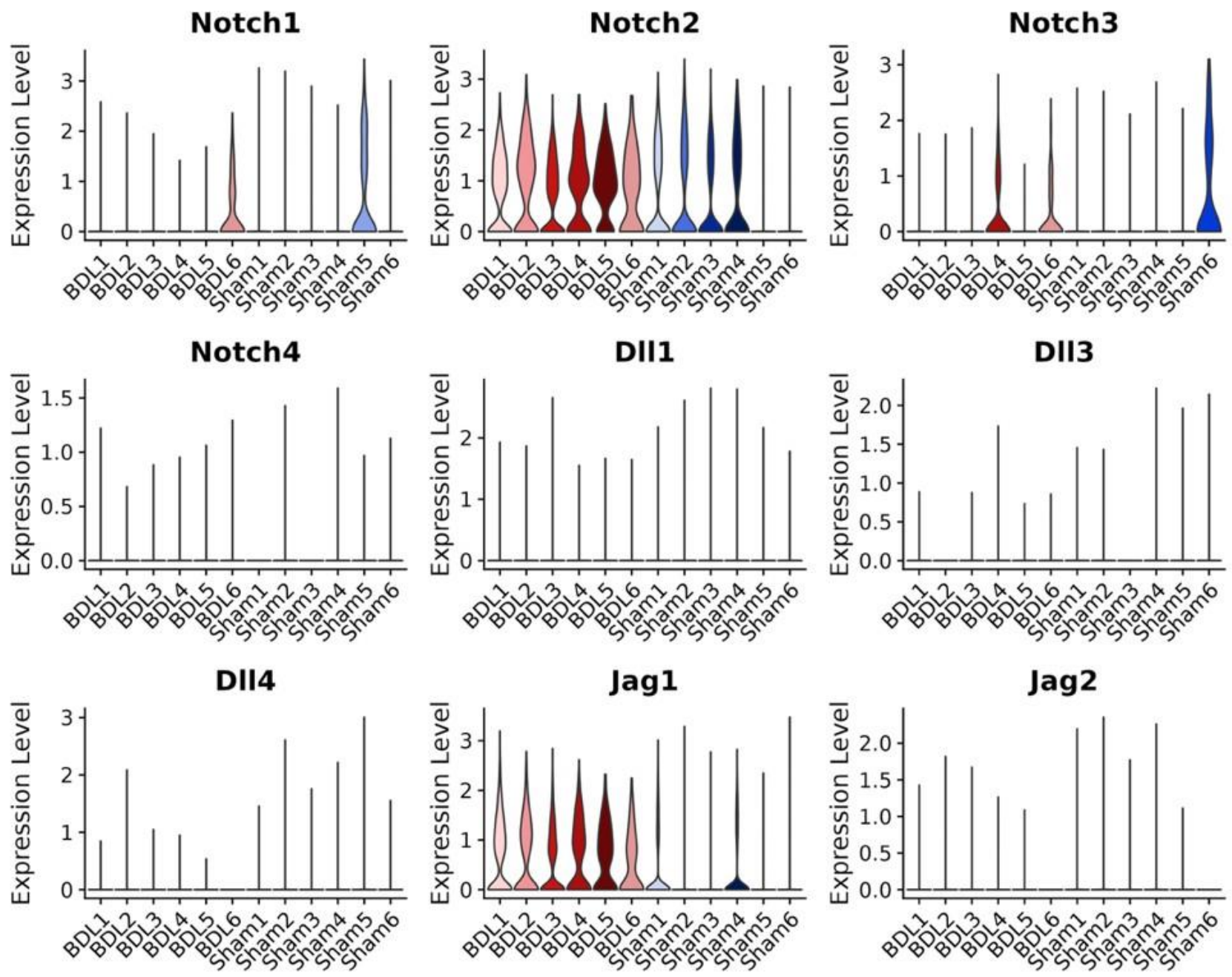

Notch1
Notch2
Notch3
Notch4
Dll1
Dll3
Dll4
Jag1
Jag2
Expression Level
BDL1
BDL2
BDL3
BDL4
BDL5
BDL6
Sham1
Sham2
Sham3
Sham4
Sham5
Sham6

REFERENCES

Jansma, A., Yao, Y., Wolfe, J., Del Debbio, L., Beentjes, S. V., Ponting, C. P. and Khamseh, A. (2025). High order expression dependencies finely resolve cryptic states and subtypes in single cell data. *Mol Syst Biol* **21**, 173–207.

Jarman, E. J., Martinez Lyons, A., Yao, Y., Rozyczko, A., Waddell, S. H., Gradinaru, A., Olaizola, P., Davies, K., Guest, R. V., Röessler, S., et al. (2025). Epithelial state-transitions permit inflammation-induced tumorigenesis. *bioRxiv*, 2025.2006.2022.660911.

Second decision letter

MS ID#: dev.205690R1

MS TITLE: Multi-gene biomarkers reveal spatial organization and subpopulation-specific damage response in biliary epithelial cells.

AUTHORS: Kendall Kanakanui; Fransky Hantelys; Hannah Hrncir; Sergei Bombin; Adam D Gracz

Dear Dr Gracz,

I am happy to tell you that your manuscript has been accepted for publication in Development, pending our standard publication integrity checks.

Reviewer 1

The authors have addressed all of my comments. I do not have any remaining concerns about this manuscript.

Reviewer 2

The authors have responded to all of my initial points. They have provided extra data where appropriate and indeed have explored alternative methods (although not included them in the manuscript as I appreciate this opens more questions than it closes).

Reviewer 3

SUMMARY OF THE ADVANCE MADE IN THIS PAPER AND ITS POTENTIAL SIGNIFICANCE TO THE FIELD

As a repeat from original review, "The information provided in this resource article is crucial to the general field of cell lineage heterogeneity and specifically to liver injury repair/response involving intrahepatic biliary epithelial cell (BEC) biology. The deeply characterized BEC single cell atlas provided in this manuscript will be unique for truly normal liver with the added and compared experimental bile duct ligation (BDL) injury response BEC single cell atlas. The authors not only took advantage of their Sox9EGFP+ mouse line to isolate BECs and thereby critically increase the number of BECs interrogated, but also used existing omic platforms in unique ways, and importantly compared and discussed published human and mouse data sets defining four BEC subtypes. Finally, the authors use HCR FISH to spatially localize their identified metagene-defined BEC subtypes in vivo and in vitro. Therefore, the two approaches and information provided in this resource article greatly strengthens the quality and rigor of the manuscript."

© 2026. Published by The Company of Biologists under the terms of the Creative Commons Attribution License (https://creativecommons.org/licenses/by/4.0/).

The revisions, using different analysis platforms - have clarified, supported, and extended the conclusions from the initial submission. Futhermore, the authors used their existing data to address controversial material in the intrahepatic biliary field (differences in molecular characterization of duct lumenal size versus size of the BEC itself) in an unbiased approach. Congratulations to the authors on their transparent responses to the reviewers' comments and requests, strengthening the value of the manuscript as a resource article.

© 2026. Published by The Company of Biologists under the terms of the Creative Commons Attribution License (https://creativecommons.org/licenses/by/4.0/).
